# CRISPR screening by AAV episome-sequencing (CrAAVe-seq): a scalable cell-type-specific in vivo platform uncovers neuronal essential genes

**DOI:** 10.1038/s41593-025-02043-9

**Published:** 2025-08-22

**Authors:** Biswarathan Ramani, Indigo V. L. Rose, Noam Teyssier, Andrew Pan, Spencer Danner-Bocks, Tanya Sanghal, Lin Yadanar, Ruilin Tian, Keran Ma, Jorge J. Palop, Martin Kampmann

**Affiliations:** 1 Institute for Neurodegenerative Diseases; Weill Institute for Neurosciences, University of California, San Francisco, San Francisco, CA USA; 2 Department of Pathology, University of California, San Francisco, San Francisco, CA USA; 3 Neuroscience Graduate Program, University of California, San Francisco, San Francisco, CA USA; 4 Biological and Medical Informatics Graduate Program, University of California, San Francisco, San Francisco, CA USA; 5 Biophysics Graduate Program, University of California, San Francisco, San Francisco, CA USA; 6 Gladstone Institute of Neurological Disease, San Francisco, CA USA; 7 Department of Neurology, University of California, San Francisco, San Francisco, CA USA; 8 Department of Biochemistry and Biophysics, University of California, San Francisco, San Francisco, CA USA

**Keywords:** Functional genomics, Molecular neuroscience, Genetics of the nervous system, High-throughput screening

## Abstract

There is a substantial need for scalable CRISPR-based genetic screening methods that can be applied in mammalian tissues in vivo while enabling cell-type-specific analysis. Here we developed an adeno-associated virus (AAV)-based CRISPR screening platform, CrAAVe-seq, that incorporates a Cre-sensitive sgRNA construct for pooled screening within targeted cell populations in mouse tissues. We used this approach to screen two large sgRNA libraries, which collectively target over 5,000 genes, in mouse brains and uncovered genes essential for neuronal survival, of which we validated *Rabggta* and *Hspa5*. We highlight the reproducibility and scalability of the platform and show that it is sufficiently sensitive for screening in a restricted subset of neurons. We systematically characterize the impact of sgRNA library size, mouse cohort size, the size of the targeted cell population, viral titer, and coinfection rate on screen performance to establish general guidelines for large-scale in vivo screens.

## Abstract (web summary)

The authors developed an adeno-associated virus-based high-throughput in vivo CRISPR screening platform for endogenous mouse brain cell types. Using this platform, they define genes and pathways essential for neuronal survival.

## Main

CRISPR-based genetic screens enable the powerful and massively parallel interrogation of gene function for biological discovery. Most CRISPR screens are carried out in cultured cells. First applications in iPSC-derived neurons^1,2^, microglia^3^ and astrocytes^4^ have uncovered cell-type-specific mechanisms relevant to neuroscience and neurological diseases. However, cultured cells do not fully recapitulate the physiological context of a multicellular organism, nor do they accurately represent tissue states such as aging, inflammation or disease. These limitations are particularly evident in applications to biological questions related to complex organs, such as the brain, which involves intricate spatial interactions between numerous distinct cell types. Therefore, in vivo pooled CRISPR screens on endogenous cells of the mouse brain have the potential to uncover insights that would be elusive in cell culture.

**Extended Data Fig. 1:**
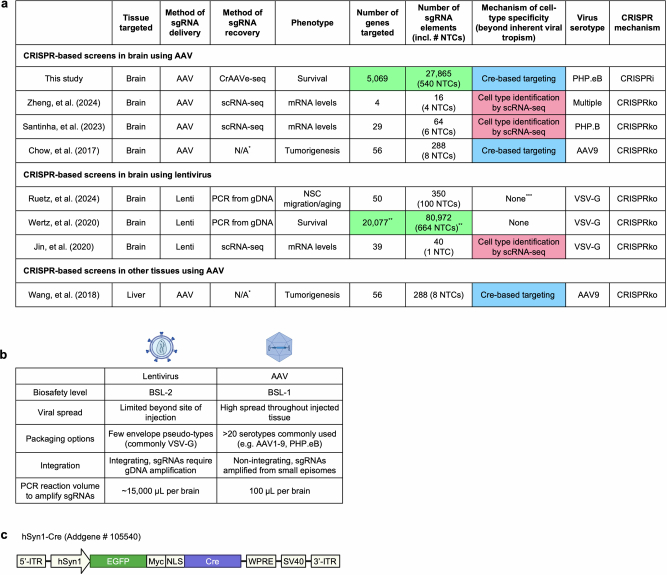
CrAAVe-seq enables simultaneously highly scalable and cell-type-specific in vivo screens. **a**, Table summarizing published in vivo pooled screens of endogenous cells in the brain and AAV-based screen outside of the brain^5–10^^,39^. *Did not capture sgRNAs, instead performed mutagenesis screen; **murine genome-wide Asiago library; ***a specific brain region containing migrated neural stem cell (NSCs) provided selectivity. CRISPRko: CRISPR knockout. Highlighted cells are large-scale sgRNA libraries (green), cell-type selectivity by Cre (blue), and single-cell RNA-sequencing (scRNA-seq, red). **b**, Table comparing different aspects of AAV and lentivirus. Virus diagrams in panel **b** are created with BioRender.com. **c**, Simplified diagram of the hSyn1-Cre plasmid construct (Addgene, 105540). Panel **c** is created with BioRender.com.

A small number of in vivo CRISPR screens targeting endogenous brain cells have previously been reported^5–10^ (reviewed in ref. ^11^; summarized in Extended Data Fig. 1a). Some of these screens involved delivering single-guide RNA (sgRNA) libraries to the brain via lentivirus, which has several drawbacks including poor distribution of lentivirus through the brain and the inability to differentiate between cell types in which specific sgRNAs were expressed (summarized in Extended Data Fig. 1b). To overcome some of these limitations, emerging studies have turned to adeno-associated virus (AAV) for more widespread brain transduction, combined with CRISPR perturbations and single-cell RNA sequencing (AAV-Perturb-Seq)^9,10^ providing granular details on transcriptional changes in specific cell types. However, the current cost of scaling this strategy to study larger cellular populations in the mouse brain and across multiple independent mice is prohibitive. Prior work using this approach has so far not exceeded a library size of 65 sgRNAs (targeting 29 genes) and a sampling of 60,000 cells^10^ (Extended Data Fig. 1a). Screens at such a scale enable the phenotypic profiling of a small number of preselected genes of interest, but not the unbiased discovery of unexpected genes that have a phenotype of interest. As such, there is a great need for a substantially more scalable in vivo CRISPR screening platform that retains the ability to discriminate between cell types.

Here we developed a strategy for screening in the mouse brain called CRISPR screening by AAV episome sequencing (CrAAVe-seq). Incorporating a Cre recombinase-based genetic element into the sgRNA library backbone enables the selective evaluation of phenotypes caused by genetic perturbations only in cell types of interest. Furthermore, CrAAVe-seq exploits the amplification of sgRNA sequences from AAV episomes^12,13^, rather than genomic DNA, to dramatically increase the scalability and reduce the cost of quantifying sgRNA frequencies from whole-brain homogenates. Using CrAAVe-seq, we profiled neuron-essential genes in the mouse brain across different neuronal subpopulations, using libraries containing ~12,000 and ~18,000 sgRNAs per brain and sampling at least 2.5 million neurons per brain. This approach yielded highly reproducible top hits across independent mice. Therefore, CrAAVe-seq enables high-throughput, cost-effective screening of the mammalian CNS, with immediate applicability to other cell types and tissues. A companion protocol for implementing CrAAVe-seq is available in ref. ^14^.

## Results

### CrAAVe-seq plasmid design and Cre-dependent sgRNA recovery

**Fig. 1:**
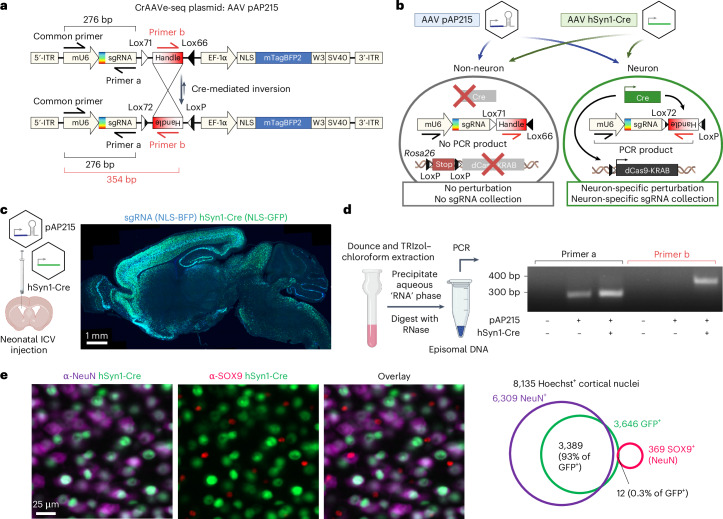
CrAAVe-seq strategy for cell-type-specific in vivo CRISPR screening using Cre-sensitive sgRNAs. **a**, Structure of CrAAVe-seq plasmid pAP215 (sgRNA backbone), which enables cell-type-specific CRISPR screening by AAV episome sequencing (CrAAVe-seq) in vivo. pAP215 expresses sgRNAs under an mU6 promoter and contains a Lox-flanked handle that inverts upon exposure to Cre recombinase. The plasmid also expresses a nuclear-localized BFP for visualization. **b**, Example of a strategy for CRISPRi screening in neurons using pAP215 and a Cre recombinase driven by a pan-neuronal hSyn1 promoter. Expression of Cre induces both expression of CRISPRi machinery (via recombination of LoxP sites of the Lox-Stop-Lox (LSL)-CRISPRi transgene in the endogenous *Rosa26* locus) and inversion of the pAP215 ‘handle’ element, which contains a primer site for PCR amplification on recovered episomes. **c**, Distribution and expression of AAV pAP215 (sgRNA, nuclear BFP^+^) and AAV hSyn1-Cre-NLS-GFP (hSyn1-Cre, nuclear GFP^+^), both packaged using PHP.eB capsid, in a mouse brain three weeks after neonatal ICV delivery. Scale bar, 1 mm. **d**, PCR performed on episomes recovered from mouse brains expressing pAP215 with or without hSyn1-Cre using the primers diagrammed in **a**. PCR amplification with primer a is invariant to Cre expression, whereas amplification with primer b requires Cre expression. Panels **a**–**d** are created with BioRender.com. **e**, Immunofluorescent staining for neurons (NeuN^+^) and astrocytes (SOX9^+^) in frontal cortex of mice injected with PHP.eB::hSyn1-Cre. Scale bar, 25 µm. Overlap of Cre expression with NeuN and Sox9 within a cortical region demonstrates high coverage and specificity for neurons. Source data

We aimed to leverage the high CNS tropism and infectivity for neurons of AAV, particularly in comparison to lentivirus, to develop an AAV-based system for pooled CRISPR perturbations of endogenous neuronal genes in the mouse brain. However, because of the ability of AAV to transduce many different cell types^15^, we sought a cell-type-specific approach for CRISPR screening. To address this, we designed pAP215, an AAV vector for sgRNA expression that contains an mU6-driven sgRNA sequence followed by a Lox71/Lox66-flanked 175 bp ‘handle’ cassette that undergoes predominantly unidirectional^16^ inversion in cells expressing Cre recombinase (Fig. 1a). The construct expresses a nuclear-localized blue fluorescent protein (NLS-mTagBFP2) for visualization and is available on Addgene (217635).

We devised a strategy using pAP215 to screen for genes essential for neuronal survival in the mouse brain in vivo (schematized in Fig. 1b). In addition to uncovering genes required for neuronal survival in a healthy brain, neuronal survival screens can be readily applied to various mouse models of neurological diseases to identify genetic drivers or modifiers of neuronal susceptibility. AAVs containing pAP215 and promoter-driven Cre recombinase (for example, a pan-neuronal hSyn1 promoter-driven Cre) are co-injected into the brains of inducible CRISPR interference (Lox-Stop-Lox-dCas9-KRAB or LSL-CRISPRi) mice^17^. Cre drives both dCas9-KRAB expression and inversion of the handle sequence. PCR amplification by priming against the inverted handle on AAV episomes, followed by sequencing, enables the identification and quantification of sgRNAs in Cre-expressing neurons.

**Extended Data Fig. 2:**
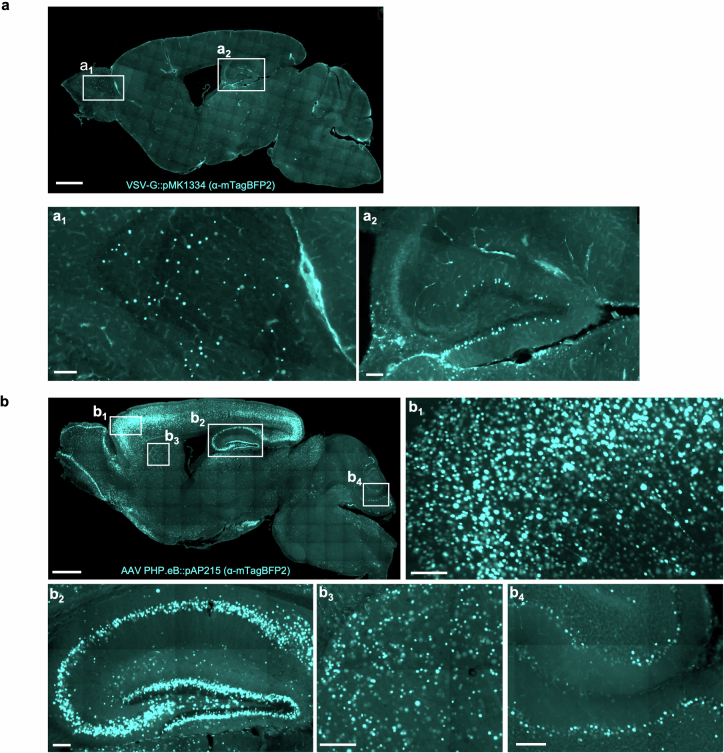
Expression of lentivirus and AAV in the mouse brain. **a**, Sagittal section of mouse brain injected with sgRNA construct packaged in VSV-G-pseudotyped lentivirus (EF1a-NLS-mTagBFP2; pMK1334), stained with antibody recognizing mTagBFP2 at 3 weeks after ICV injection. Scale bar is 1 mm for low-magnification and 100 µm for subpanels (a1) and (a2). **b**, Sagittal section of mouse brain injected with sgRNA construct packaged in AAV PHP.eB capsid (EF1a-NLS-mTagBFP2; pAP215), stained with antibody recognizing mTagBFP2 at 3 weeks after ICV injection. This condition is sgCreb1 + hSyn1-Cre (mouse 2), used in Fig. 2 and in Extended Data Fig. 4. Scale bar is 1 mm for low magnification and 100 µm for subpanels (b1), (b2), (b3), and (b4).

We packaged our AAV plasmids into the PHP.eB capsid, which is known to enable widespread transduction of brain cells^18^. We co-injected pAP215 (PHP.eB::pAP215) and an hSyn1 promoter-driven Cre recombinase tagged with a nuclear GFP (PHP.eB::hSyn1-Cre; diagram of construct shown in Extended Data Fig. 1c) by intracerebroventricular (ICV) injection into neonatal mice. We observed a broad distribution of both pAP215 and hSyn1-Cre reporters across the brain, particularly in the cortex and hippocampus (Fig. 1c), consistent with the known distribution profile of the PHP.eB capsid^19^. Contrasting this, we observe much more limited expression and spread of the same fluorescent reporter delivered by lentivirus (Extended Data Fig. 2).

We performed isopropanol precipitation of nucleic acids from the aqueous phase of a TRIzol-chloroform extraction of an AAV-injected whole mouse brain, followed by resuspension in 50 µl water with RNase. pAP215 can be detected in this ‘episome’ fraction by PCR (Fig. 1d). Notably, we found that the Cre-inverted handle sequence was detected only in mice co-injected with hSyn1-Cre (Fig. 1d). Furthermore, the entire episome fraction can be used in a single 50–100 µl PCR reaction volume, markedly expanding scalability of sample preparation for sgRNA sequencing in the context of a pooled screen, as compared to lentiviral approaches necessitating PCR amplification from the entire genomic DNA (detailed in Supplementary Information).

PHP.eB::hSyn1-Cre (expressing nuclear GFP) essentially only expresses in NeuN^+^ neurons, and not SOX9^+^ astrocytic nuclei (Fig. 1e), confirming hSyn1 is neuron specific^20^. Of note, while 0.3% of GFP overlapped with SOX9^+^ nuclei based on automated analysis, manual inspection of these nuclei showed GFP signal was emanating from adjacent neurons without observable distinct nuclear GFP signal within SOX9^+^ nuclei.

### CRISPRi knockdown in vivo using AAV

**Fig. 2:**
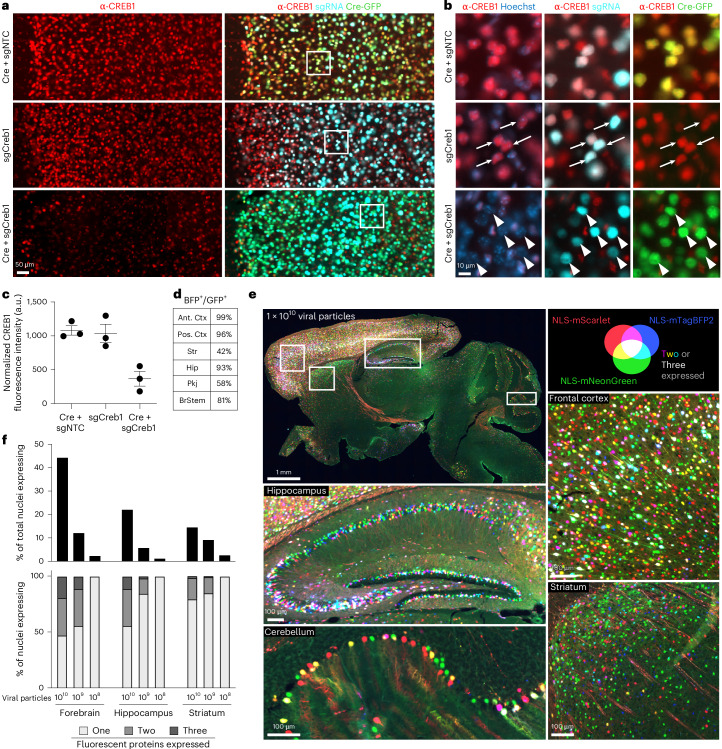
Cre-dependent CRISPRi knockdown of CREB1 in vivo and estimating extent of AAV multiple infections. **a**, sgRNAs targeting the nonessential gene *Creb1* (sgCreb1) or a nontargeting control (sgNTC) were cloned into pAP215 and PHP.eB-packaged, then delivered with or without PHP.eB::hSyn1-Cre by ICV injection into neonatal LSL-CRISPRi mice. Brains were stained at 3 weeks to determine reduction in CREB1 levels; a representative area of frontal cortex is shown, with cortical layer 1 oriented to the left. Scale bar, 50 µm. Other brain regions are shown in Extended Data Fig. 3. Images with higher brightness are used to identify cells with low GFP or BFP expression. **b**, Higher magnification of the boxed regions in panels from **a**, with arrowheads indicating examples of neurons having received both sgCreb1 and Cre, whereas arrows indicate neurons that received Cre only or sgCreb1 only. Scale bar, 10 µm. **c**, Quantification of CREB1 levels in sgRNA-containing (BFP^+^) nuclei within a representative region of cortex (mean ± s.e.m, *n* = 3 independent mice). Other brain regions are quantified in Extended Data Fig. 4. **d**, Table showing the percentage of neurons in a sgCreb1 + Cre mouse 2 that are positive for both BFP and GFP, and in different brain regions. **e**, To estimate the number of multiple infections, AAV (PHP.eB serotype) expressing nuclear-localized mNeonGreen, mTagBFP2, or mScarlet fluorescent proteins were copackaged and co-injected by ICV into neonates at three different concentrations, and the highest concentration (1 × 10^10^ viral particles per mouse) is shown here across multiple brain regions. Lower concentrations are shown in Extended Data Fig. 5. **f**, Top, quantification of data from **e** and Extended Data Fig. 5, showing percentage of nuclei that were expressing at least one fluorescent protein in the forebrain, hippocampus and striatum at three different concentrations of injected AAV (1 × 10^10^, 10^9^, and 10^8^ viral particles per mouse). Bottom, percent of nuclei expressing one, two, or all three fluorescent proteins for the same experiments. Ctx, cortex; Pkj, Purkinje; Hippo, hippocampus; BrStem, brainstem; Str, striatum.

**Extended Data Fig. 3:**
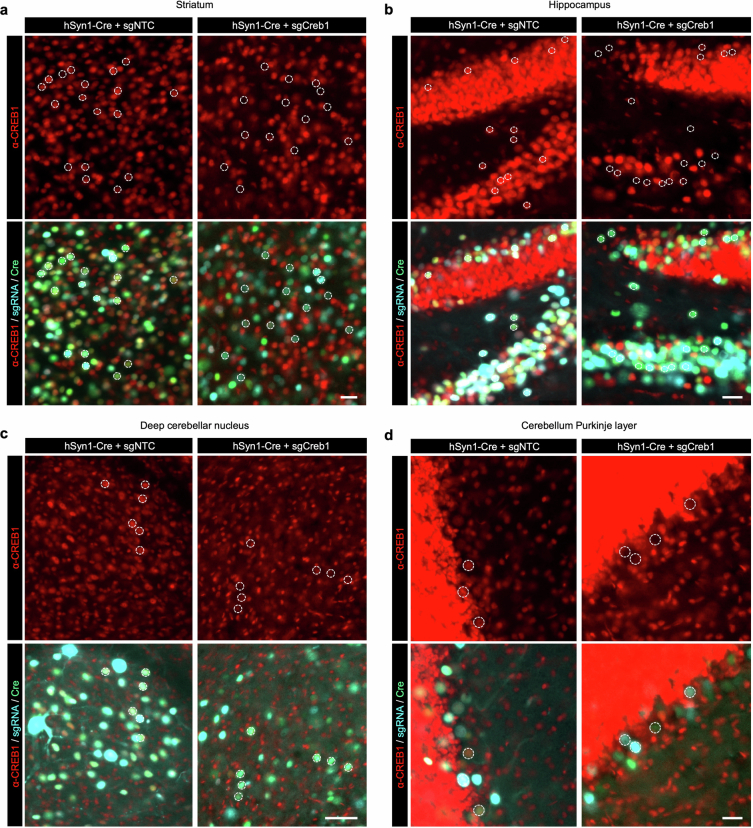
Cre-dependent CRISPRi knockdown of CREB1 in different brain regions of a mouse. Immunofluorescent staining for CREB1 (red) in an LSL-CRISPRi mouse 3 weeks after injection with PHP.eb::pAP215-sgNTC or -sgCreb1 (blue) along with PHP.eB::hSyn1-Cre (green), for different brain regions (**a**, striatum; **b**, hippocampus; **c**, deep cerebellar nuclei; **d**, cerebellum Purkinje layer). Representative nuclei containing both BFP and GFP are outlined with a dotted circle, with the same nuclei shown in the top and bottom panels of each brain region, highlighting markedly reduced CREB1 in double-positive nuclei with sgCreb1 as compared to sgNTC. DG: dentate gyrus; ML: molecular layer, Pkj: Purkinje cell layer (Pkj); IGL: internal granule layer. Data is quantified in Extended Data Fig. 4b. Scale bars are 20 µm in a–d.

**Extended Data Fig. 4:**
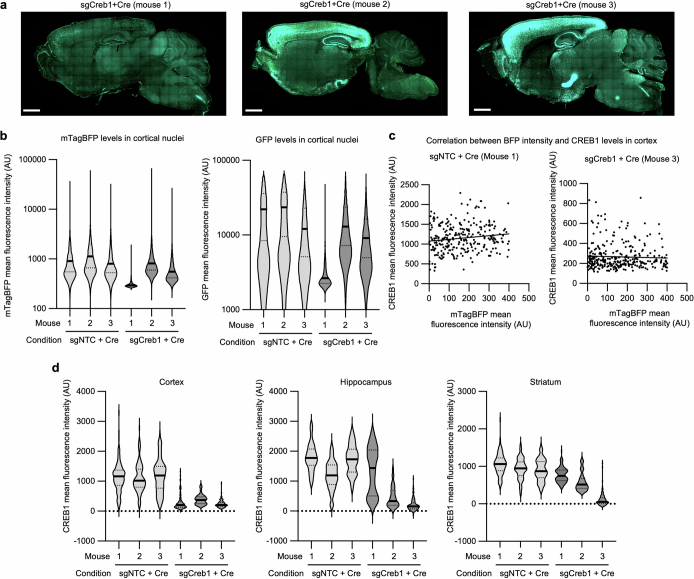
Quantification of CREB1 by brain region and by BFP intensity. **a**, Sagittal sections of the three mice injected with AAV pAP215 expressing sgCreb1 and AAV hSyn1-Cre (Cre) that are used in Fig. 2. Mouse 1 has a lower expression of both the sgRNA and Cre, potentially due to injection variability. Scale bars are 1 mm. **b**, Quantification of levels of BFP and GFP in a representative region of the cortex for sgNTC + Cre and sgCreb1 + Cre mice, n = 3 each. **c**, CREB1 levels plotted against BFP levels in a representative region of cortex from two different mice, with trendlines generated by simple linear regression showing no correlation between BFP and CREB1 levels. **d**, Quantification of CREB1 levels in BFP+ nuclei of three different brain regions.

To test whether the pAP215 plasmid is effective for CRISPRi knockdown, we used an sgRNA targeting *Creb1* (sgCreb1) in comparison to a nontargeting sgRNA (sgNTC). *Creb1* encodes a ubiquitous but nonessential nuclear protein^21^. We co-injected PHP.eB::pAP215-sgCreb1 or PHP.eB::pAP215-sgNTC alongside PHP.eB::hSyn1-Cre by ICV into neonatal LSL-CRISPRi mice, at approximately 1 × 10^11^ viral particles (vp) per mouse. Three weeks after neonatal ICV injection, immunofluorescence staining confirmed strong knockdown of endogenous CREB1 in all neurons that received both sgCreb1 (BFP^+^ nuclei) and hSyn1-Cre (GFP^+^ nuclei; Fig. 2a–c and Extended Data Figs. 3 and 4). In contrast, neurons that received sgCreb1 alone did not show knockdown, indicating no apparent leakiness of dCas9-KRAB expression. We also observed that >90% of cortical and hippocampal neurons express both the Cre and the sgRNA, with less coinfection in striatum and cerebellum (Fig. 2d). This indicated that codelivery of the sgRNA and Cre viruses can provide broad coverage and CRISPRi activity for a broad distribution of brain regions. We noted that one sgCreb1+hSyn1-Cre mouse showed overall less viral transduction throughout the brain, most likely due to a technical issue during the virus injection (mouse 1). However, even with sparse infection, there was still a strong knockdown of CREB1 in all BFP^+^/GFP^+^ nuclei at the level of individual cells, suggesting that transduction by an AAV at low MOI (potentially a single sgRNA) is sufficient for knockdown. Furthermore, we found no correlation between BFP levels and the degree of CREB1 knockdown in a brain transduced with greater amounts of sgCreb1 + hSyn1-Cre (mouse 3; Extended Data Fig. 4c).

### Distribution and extent of multiple infections by AAV

The high degree of coinfection between the sgRNA and Cre above in most mice prompted us to obtain a semiquantitative estimate of multiplicity of infection (MOI) by injecting AAVs at multiple concentrations. We copackaged equimolar concentrations of AAV plasmids encoding three different nuclear-localized fluorescent proteins and performed neonatal ICV injections using three different total viral particle amounts per mouse—1 × 10^10^, 1 × 10^9^ and 1 × 10^8^. Three weeks postinjection, we imaged representative sagittal brain sections to evaluate the prevalence of single-, double- and triple-infected nuclei across different brain regions.

**Extended Data Fig. 5:**
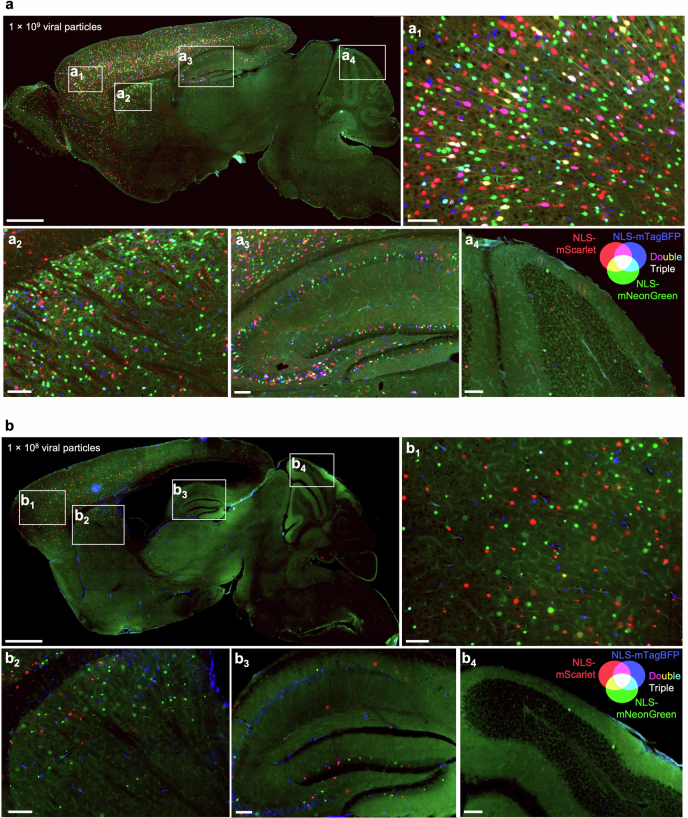
Distribution and extent of multiple infections with AAV injected at lower concentrations. Sagittal sections of mouse brains at 3 weeks after ICV co-injection of PHP.eB-packaged AAV expressing nuclear-localized mScarlet, mNeonGreen, or mTagBFP, at the indicated viral concentrations, which are (**a**) 10-fold or (**b**) 100-fold lower than show in Fig. 2. Scale bars: 1 mm for low-magnification views and 100 µm for subpanels.

Even at the highest concentration, most nuclei expressed only one or two fluorescent proteins, with approximately 15% of nuclei in the densely transduced forebrain showing infection with all three fluorescent proteins (Fig. 2e,f). Regions with higher coinfection rates correlated directly with areas of enhanced viral tropism. At lower concentrations, there was a sharp decline in the total number of transduced nuclei, accompanied by a higher fraction of nuclei expressing only one type of fluorescent protein (Fig. 2f and Extended Data Fig. 5). In short, the results indicated that higher viral concentrations maximized the number of transduced neurons, and that even at 1 × 10^10^ viral particles per brain, a substantial number of cells in most brain regions express a single type of fluorescent protein, indicating that most of these cells are transduced by a single virus (with presumably a small fraction of these representing coinfection by two viruses expressing the same fluorescent protein). Obtaining a precise MOI from these experiments is challenging due to the variability of infection between different brain regions and neuronal types as dictated by viral tropism. Despite this limitation, these findings help us broadly estimate that the MOI across the whole brain injected with 1 × 10^10^ viral particles most likely ranges from 1 to 3, and could be higher than 3 in focal areas of strong tropism.

### CrAAVe-seq identifies neuron-essential genes

**Fig. 3:**
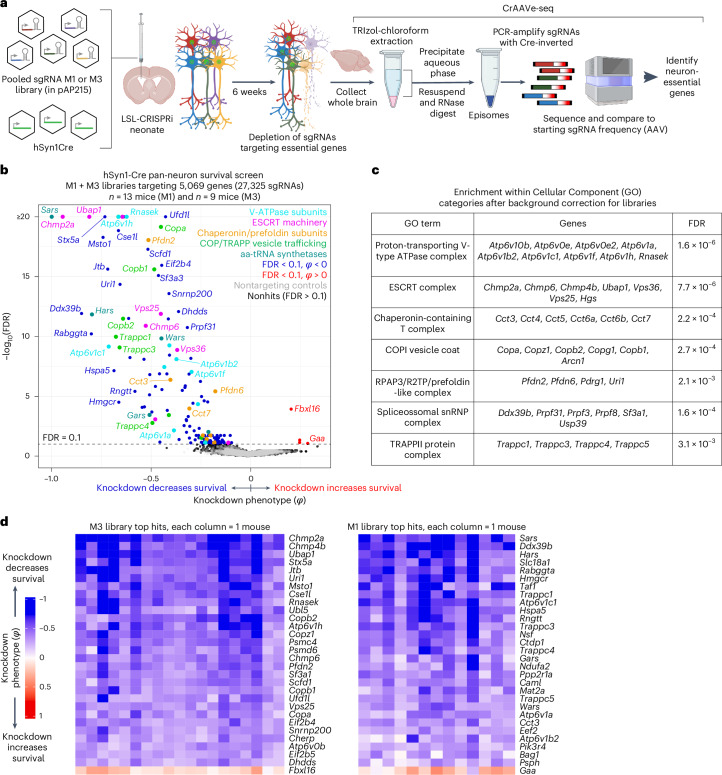
In vivo CrAAVe-seq uncovers neuron-essential genes in the mouse brain. **a**, Experimental design for CrAAVe-seq to identify neuron-essential genes across two different pooled sgRNA libraries (pAP215 expressing the M1 or M3 libraries). M1 targets kinases, phosphatases and druggable targets—2,269 genes (12,100 targeting sgRNAs and 250 nontargeting controls); M3 targets stress and proteostasis targets—2,800 genes (14,685 targeting sgRNAs and 290 nontargeting controls)^22^. The sgRNA libraries and hSyn1-Cre were packaged into AAV (PHP.eB capsid) and co-injected into neonatal LSL-CRISPRi mice. Panel **a** is created with BioRender.com. **b**, Results from CRISPRi screen described in **a**—knockdown phenotype (φ; Methods) for 5,069 genes and nontargeting (quasi-gene) controls at 6 weeks postinjection (*n* = 13 mice injected with pAP215-M1 library, and *n* = 19 mice injected with pAP215-M3 library). Hit genes (FDR < 0.1) within enriched pathways are color coded. Nontargeting controls (gray) are plotted on top of nonhit genes (black). **c**, Table showing enrichment of hit genes in different Cellular Component GO terms. **d**, Knockdown phenotype for each individual mouse (columns) for 30 top hit genes (rows, 29 with negative and 1 with positive knockdown phenotypes). Rows are ranked by average knockdown phenotype. GO, Gene Ontology.

We generated two different sgRNA pooled libraries in the pAP215 AAV backbone by transferring sgRNAs from our previously established mouse sgRNA libraries^22^. This includes the ‘M1’ library that contains 12,350 sgRNAs targeting 2,269 genes, including kinases, phosphatases and other druggable targets, and the ‘M3’ library containing 14,975 sgRNAs targeting 2,800 proteostasis and stress genes^22^. The M1 and M3 libraries contain 250 and 290 sgNTC, respectively. We codelivered PHP.eB::hSyn1-Cre alongside either PHP.eB::pAP215-M1 library (n = 13 mice) or PHP.eB::pAP215-M3 library (*n* = 19 mice) by ICV injection into LSL-CRISPRi mice. We collected the brains after 6 weeks, recovered episomes and amplified sgRNAs using primers specific to the Cre-inverted handle in pAP215, followed by sequencing (Fig. 3a). We compared the sgRNA frequencies from the recovered episomes with those of the viral preparation used for injections. We refer to this workflow as ‘CrAAVe-seq’ throughout the rest of this study. The age, sex and sgRNA read depth for each mouse are provided in Supplementary Table 1.

**Extended Data Fig. 6:**
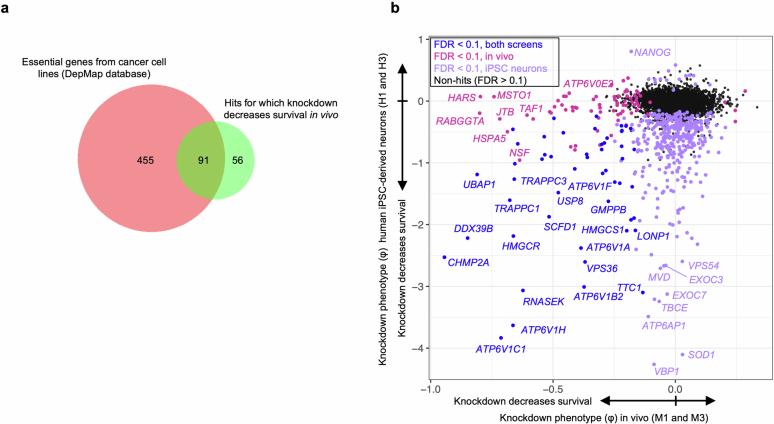
Overlap of hits of in vivo screens with essential genes of iPSC-derived neurons, and DepMap common essential genes. **a**, Venn diagram showing that 91 of the 147 hit genes for which knockdown decrease neuronal survival in vivo (Fig. 3) overlap with common essential genes of the injected libraries in cancer cell lines described in the DepMap database^23^, P < 0.0001 by Fisher’s exact test, two-sided. **b**, Comparison of knockdown phenotype for genes between the in vivo neuronal survival screen of Fig. 3 and survival screens previously performed in iPSC-derived neurons by ref. ^2^.

Analysis of changes in sgRNA frequencies using an optimized computational pipeline (see Methods for details) identified 147 genes with significant sgRNA depletion (false discovery rate (FDR) < 0.1), indicating that knockdown of these genes promoted neuronal death (Fig. 3b). The output knockdown phenotype of all screens is provided in Supplementary Table 2. Gene-set enrichment analysis (with background correction for the genes in the library) further highlighted that many hit genes belonged to distinct biological categories (Fig. 3c), such as the vacuolar ATPase complex, ESCRT pathways, COP vesicle trafficking and the chaperonin/TRiC complex. Although not identified through gene enrichment, we also noted that some of the top hits were aminoacyl tRNA synthetase genes. Supporting that the genes with a negative knockdown phenotype are essential, we found that 91 of the 147 hit genes (62%) overlapped with known common essential genes established by DepMap from cancer cell lines^23^ (Extended Data Fig. 6a). We similarly found substantial overlap with our prior screens for essential genes performed in induced pluripotent stem cell-derived neurons^2^ (Extended Data Fig. 6b). Nonetheless, we identified some unique hits that have not been previously reported, including *Jtb*, *Snx17*, *Psph*, *Bag1* and *Becn1*. sgRNAs targeting a few genes were weakly enriched relative to input AAV, suggesting that knockdown of these genes enhances neuronal survival.

**Extended Data Fig. 7:**
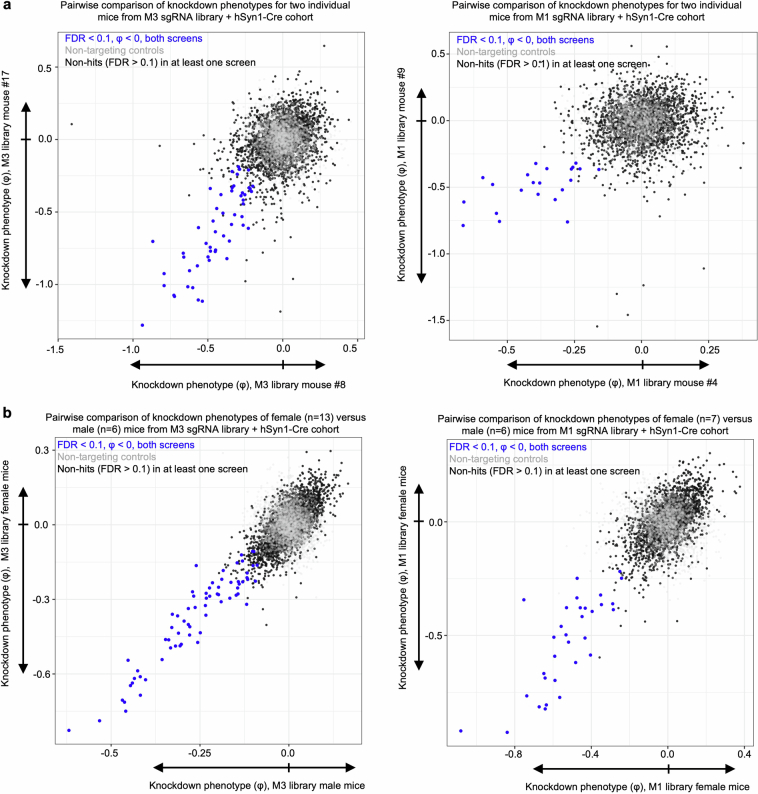
Pairwise comparisons of CRISPR screens by individual mice and by biological sex. **a**, Pairwise correlation of knockdown phenotypes between two individual mice injected with hSyn1-Cre and the M3 library (left) and the M1 library (right). The two individual mice that made the greatest number of hits in each screen were selected for these comparisons. **b**, Pairwise correlation of combined knockdown phenotypes between male and female mice injected with hSyn1-Cre and the M3 library (left) and the M1 library (right).

**Extended Data Fig. 8:**
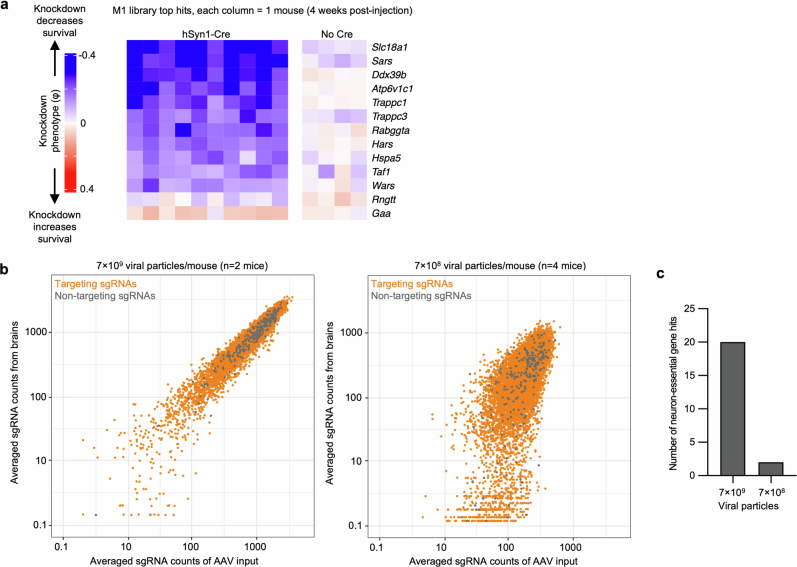
Cre expression and concentration dependency for screening performance. **a**, Heatmap of a subset of top hit genes in LSL-CRISPRi mice at 4 weeks after injection of PHP.eB::pAP215-M1 library, with or without co-delivery of PHP.eB::hSyn1-Cre. **b**, A cohort of LSL-CRISPRi mice were injected with either 7 × 10^9^ (n = 2 mice) or 7 × 10^8^ (n = 4 mice) viral particles of the PHP.eB::pAP215-M1 library in littermates. Normalized and averaged sgRNA counts from the brains are plotted against the sgRNA counts from the input AAV library, showing marked dropout of both targeting and nontargeting sgRNAs when using the lower concentration of virus. **c**, Number of neuron-essential genes identified based on overlap with hits identified from the M1 library cohort in Fig. 3b in the mice with different concentrations of virus.

To examine the robustness of our screening approach, we compared knockdown phenotypes for top hits from each library in each mouse and found that phenotypes were highly reproducible across individual mice (Fig. 3d), corroborating that hits obtained from analyzing the whole cohort of mice are not entirely driven by a subset of mice. Knockdown phenotypes were strongly correlated, particularly for the strongest hit genes between the two individual mice that identified the most hits within each cohort (Extended Data Fig. 7a). The knockdown phenotypes also strongly correlated when comparing male versus female mice from each cohort; no sex-specific neuron-essential genes were observed (Extended Data Fig. 7b). We further confirmed that the knockdown phenotypes of the top hit genes in the M1 library required Cre expression (Extended Data Fig. 8a). The knockdown phenotypes for all genes per individual mouse are provided in Supplementary Table 3.

The amount of injected AAV library for the M1 and M3 screens above were 2 × 10^10^ and 5 × 10^10^ viral particles, respectively, per mouse. Based on our MOI experiments (Fig. 2d,e), these concentrations are expected to transduce neurons in several brain regions with an MOI greater than 1, leading to the expression of multiple different sgRNAs within some neurons. To investigate the impact of MOI on screen quality, we injected a cohort of LSL-CRISPRi littermates with PHP::hSyn1-Cre along with one of two different lower concentrations of the PHP.eB::pAP215-M1 library. Evaluating two mice injected with an intermediate amount (7 × 10^9^ viral particles) of the library showed a clear, strong correlation of the sgRNAs frequencies recovered from mouse brains with those in the input AAV library (Extended Data Fig. 8b). In contrast, four mice injected with a tenfold lower concentration (7 × 10^8^ viral particles) showed much poorer correlation and extensive sgRNA dropout. The intermediate concentration identified 20 hits that overlap with hits of the M1 sgRNA library cohort from Fig. 3b, while the lower concentration identified just 2 hits meeting these criteria (Extended Data Fig. 8c). In summary, although the concentrations used for the original screens have an MOI greater than 1 in several brain regions, injecting smaller amounts of virus dramatically reduces the power of the screen. We suggest that the poor performance at lower viral concentrations is due to reduced coverage, as indicated by the vastly increased sgRNA dropout at lower concentrations. Conversely, the infection of a proportion of cells with more than one sgRNA expected at higher concentrations seems to have a much less detrimental impact on the screen quality, likely because the majority of sgRNAs do not cause a phenotype, and because each individual sgRNA is screened in many independent neurons.

### Screening for essential genes in CaMKII^+^ neurons in vivo

**Fig. 4:**
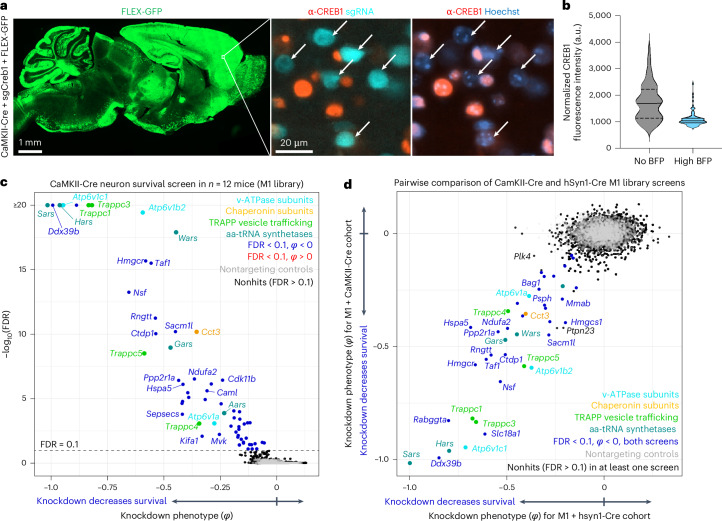
Genetic modifiers of survival in a CaMKII^+^ subpopulation of neurons in the mouse brain in vivo*.* **a**, PHP.eB::CaMKII-Cre was co-injected with PHP.eB::FLEX-GFP and PHP.eB::pAP215-sgCreb1 in LSL-CRISPRi mice. Brains were examined by immunofluorescence staining for CREB1 at 3 weeks. Left, low-power view showing expression of FLEX-GFP across a sagittal section of the brain. Middle and right, magnified region of frontal cortex showing CREB1 knockdown in BFP^+^ (sgCreb1^+^) cells (arrows). Scale bars, 1 mm (left) and 20 µm (middle). **b**, CREB1 levels in BFP^+^ (*n* = 79 cells) versus BFP^−^ (*n* = 78 cells) nuclei in a representative region of cortex. **c**, Knockdown phenotypes for 2,269 genes averaged across 12 mice at 6 weeks after ICV injection of PHP.eB-packaged M1 sgRNA library and CaMKII-Cre. Experimental design is identical to Fig. 3a, except injecting CaMKII-Cre instead of hSyn1-Cre. Genes within enriched pathways are color coded. **d**, Comparison of CRISPRi screen results between the CaMKII-Cre cohort (**c**) and the hSyn1-Cre cohort shown in Fig. 3b.

To determine if a different neuronal Cre driver produces similar findings, we tested a CaMKII promoter that has traditionally been used to target forebrain excitatory neurons^24^. Co-injecting PHP.eB::CaMKII-Cre, a LoxP-dependent GFP reporter (FLEX-GFP) and PHP.eB::pAP215-sgCreb1 into an LSL-CRISPRi mouse led to widespread GFP expression (Fig. 4a), consistent with recent reports using AAVs with CaMKII-driven reporters in the mouse brain^25,26^. In areas of the forebrain with high GFP expression, we observed a strong knockdown of CREB1 in BFP^+^ nuclei (Fig. 4a,b). We performed CrAAVe-seq in a cohort of 12 mice injected with PHP.eB::pAP215-M1 library and CaMKII-Cre, revealing a robust profile of neuron-essential genes (Fig. 4c). The knockdown phenotypes of the top hits of this CaMKII-Cre cohort strongly correlated with the hits from the hSyn1-Cre cohort of Fig. 3 (Fig. 4d), supporting that CrAAVe-seq reproducibly detects neuron-essential genes in vivo.

#### Requirement of CrAAVe-seq in a smaller neuronal population

**Fig. 5:**
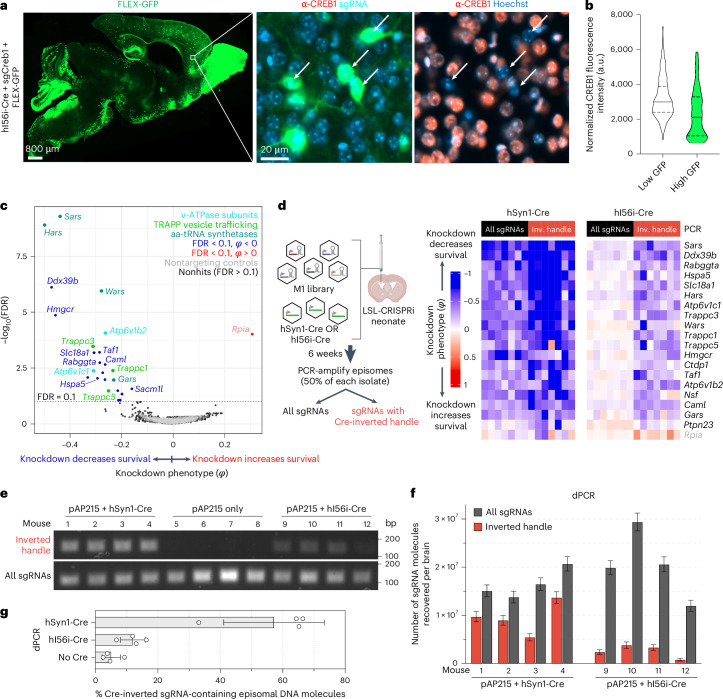
Cre-dependent sgRNA recovery is critical for screening on small neuronal subpopulations. **a**, PHP.eB::hI56i-Cre (predicted to target predominantly forebrain GABAergic interneurons)^27^ was co-injected with PHP.eB::FLEX-GFP and PHP.eB::pAP215-sgCreb1 in LSL-CRISPRi mice, followed by immunofluorescence staining for CREB1 in the brain at 3 weeks. Left, sagittal brain section showing FLEX-GFP expression (reporting Cre activity). Middle and right, inset showing CREB1 knockdown in GFP^+^ cells (arrows). Scale bars, 800 µm (left) and 20 µm (middle). **b**, CREB1 levels in GFP^+^ (*n* = 189 cells) versus GFP^−^ (*n* = 188 cells) cells within a representative cortical region. **c**, Averaged knockdown phenotypes for 2,269 genes across 11 mice, 6 weeks after ICV injection of PHP.eB::M1 sgRNA library and PHP.eB::hI56i-Cre. *Rpia* is labeled in light pink, indicating its phenotype was driven by only one sgRNA. **d**, Left, experimental design comparing the impact of PCR recovery of all sgRNAs versus only those with the Cre-inverted handle, in mice injected with M1 library and either hSyn1-Cre or hI56i-Cre. Right, heatmap showing knockdown phenotypes for top hits (rows) across *n* = 7 mice (columns) from each Cre cohort, contrasting the two PCR recovery methods. Genes listed are top hits selected from **c** and include 19 with negative and 1 with positive knockdown phenotypes. The left image in panel **d** is created with BioRender.com. **e**, Ethidium bromide-stained agarose gel for PCR products from episomes recovered from brains of mice at 4 weeks post-ICV injection. **f**, dPCR performed on Cre-injected samples in **e** showing absolute numbers of pAP215 sgRNA-encoding episomal DNA molecules recovered from each brain using CrAAVe-seq (error bars represent 95% confidence interval from dPCR Poisson distribution), both for total episomes and episomes with cell-type-specific Cre-inverted handle. **g**, Percent recovered sgRNAs from **f** that contains the inverted handle upon hSyn1-Cre (all neurons) or hI56i-Cre (small subset) expression, including no Cre-negative control (*n* = 4 mice per condition, mean ± s.d.). dPCR, digital PCR. Source data

We next wanted to evaluate whether CrAAVe-seq is sensitive enough to identify neuron-essential genes from smaller neuronal subpopulations. We selected a recently developed AAV plasmid that uses enhancer elements designed to express Cre recombinase in forebrain GABAergic neurons (CN1851-rAAV-hI56i-minBglobin-iCre-4X2C-WPRE3-BGHpA, which we refer to here as ‘hI56i-Cre’)^27^. Co-injection of this Cre alongside PHP.eB::pAP215-sgCreb1 and PHP.eB::FLEX-GFP in LSL-CRISPRi mice showed its distribution to be much more restricted than that of hSyn1-Cre and CaMKII-Cre, with a large degree of transduction within the olfactory bulb (Fig. 5a). In areas of the cortex with sparse GFP expression where individual non-overlapping nuclei could be confidently examined, GFP^+^ nuclei showed reduced levels of CREB1, again confirming Cre-dependent CRISPRi knockdown (Fig. 5b).

**Extended Data Fig. 9:**
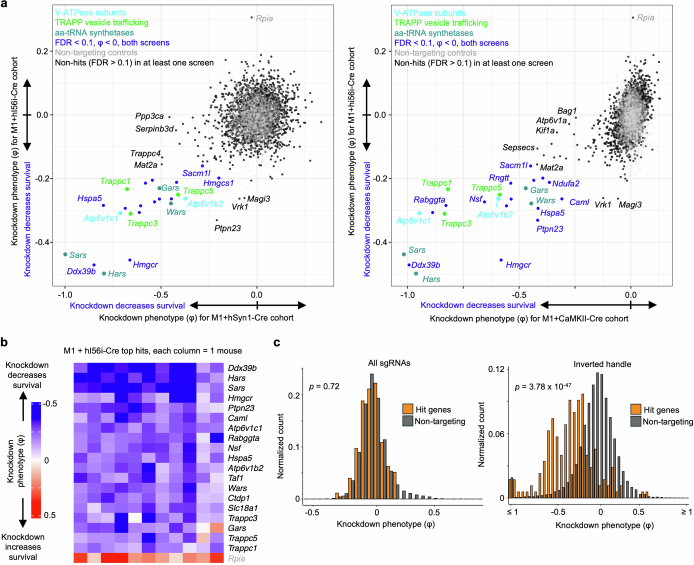
Knockdown phenotypes of essential genes identified by hI56i-Cre CRISPRi screen. **a**, Comparison of the knockdown phenotypes between the hI56i-Cre cohort and the hSyn1-Cre cohort of Fig. 3 (left) and the CaMKII-Cre of Fig. 4 (right). *Rpia* is labeled in gray to indicate that its effect is driven by only a single (but strong) sgRNA. **b**, Knockdown phenotypes plotted on a heatmap for top hit genes (rows, 19 with negative and 1 with positive knockdown phenotype) for all of the individual mice (columns). *Rpia* is shown in gray to reflect that its knockdown phenotype is driven by only one sgRNA. **c**, Histogram showing the distribution of gene knockdown phenotypes (φ) for either hit genes (orange), from (**b**), or for nontargeting control-based quasi-genes (gray) from the pAP215-M1 + hi56i-Cre screening cohort of mice shown in Fig. 5d. These plots compare the distributions for either all sgRNAs (left) or sgRNAs with the inverted handle (right). Knockdown phenotypes for each gene across the cohort were binned by 0.05 increments and normalized to the total number of genes (or quasi-genes) in that respective set. *P*-value calculated by asymptotic two-sample, two-sided Kolmogorov–Smirnov test.

We performed CrAAVe-seq on a cohort of 11 mice at 6 weeks after neonatal ICV injection of the PHP.eB::pAP215-M1 library alongside hI56i-Cre. This screen again revealed a profile of essential neuronal genes (Fig. 5c), with hits that almost fully overlapped with the hSyn1-Cre and CaMKII-Cre screens (Extended Data Fig. 9a). One exception was *Rpia*, which was uniquely identified as a hit in this hI56i-Cre cohort, but we also noted that its effect was driven by only one sgRNA out of the five targeting the gene; all other hit genes resulted from at least two significant differentially abundant sgRNAs for that gene. Nonetheless, these screens demonstrated that CrAAVe-seq can sensitively detect neuron-essential genes in a smaller neuronal population. Top hits showed consistently reproducible knockdown phenotypes in individual mice (Extended Data Fig. 9b).

To determine if the Cre-dependent recovery of sgRNA sequences is required for screening in vivo, we examined cohorts of mice injected with the PHP.eB::pAP215-M1 library together with either PHP.eB::hSyn1-Cre or PHP.eB::hI56i-Cre. However, we used half of each episome preparation for PCR amplification of all sgRNAs and the other half for PCR amplification of only those that underwent Cre-dependent inversion of the handle (Fig. 5d; primer pairs as schematized in Fig. 1a). For screens conducted with hSyn1-Cre, analyzing all sgRNAs provided knockdown phenotypes in the same direction for the hit genes in Fig. 5c, whereas analyzing only sgRNAs with the Cre-inverted handle often demonstrated a noticeable increase in the knockdown phenotypes (Fig. 5d). This indicated that hSyn1-Cre drives sgRNA activity in a sufficiently large number of neurons to permit identification of hit genes when evaluating all recovered sgRNAs, but that the presence of non-transduced or transduction in non-neuronal cells could partially mask the effects of active hits. In contrast, screens in a small neuronal subpopulation accessed by hI56i-Cre required amplification with the Cre-inverted handle to detect knockdown phenotypes (Fig. 5d). Indeed, hit genes were significantly different compared to nontargeting controls when examining sgRNAs with the Cre-inverted handle (*P* < 10^−46^), but not when examining all sgRNAs (*P* = 0.72; Kolmogorov–Smirnov test; Extended Data Fig. 9c). This supports that sgRNAs expressed in Cre-negative neurons can fully obscure the signal of active sgRNAs, making the Cre-inverting handle in pAP215 essential for screening smaller subpopulations among a broadly transduced population.

To further quantify the proportion of the total sgRNAs and Cre-inverted sgRNAs, as well as to determine their relative proportions after inversion with different Cres, we conducted PCR with different primer pairs. Episomes were recovered from mice injected PHP.eB::pAP215-M1 library alone, or with either PHP.eB::hSyn1-Cre or PHP.eB::hI56i-Cre. Standard PCR, followed by examination of the products by gel electrophoresis, showed that with primers targeting total sgRNAs, there is a qualitatively equal intensity of the PCR products. When using primers targeting the Cre-inverted handle, there was a strong band in mice injected with hSyn1-Cre, no detectable product in mice without Cre, and a weak band with hI56i-Cre (Fig. 5e).

To quantify these results, we performed digital PCR using the same primer pairs and the same samples. Furthermore, 15–30 × 10^6^ episomal DNA molecules could be recovered from each brain, with similar ranges in both Cre conditions (Fig. 5f). The number of sgRNAs containing the Cre-inverted handle in the hSyn1-Cre condition were generally a majority fraction of the total, with 5.4–13 × 10^6^ molecules detected corresponding to 57% of total sgRNAs on average (Fig. 5f,g). In contrast, with hI56i-Cre expression we detected far fewer molecules, 0.8–3.8 × 10^6^ molecules containing the inverted handle, corresponding to 12% of the total sgRNAs (Fig. 5f,g), consistent with findings on the DNA gel in Fig. 5e. In comparison, the no-Cre controls generated around 5% signal for inverted PCR primer pair, which we interpret as technical background.

Overall, these results indicate that CrAAVe-seq is extremely sensitive and is critical for screening on neuronal subpopulations. Moreover, the number of molecules detected also demonstrates the substantially large number of neurons being sampled per mouse. When performing CrAAVe-seq using hSyn1-Cre, we estimate from the above digital PCR experiments that 5 million Cre-inverted sgRNA molecules at minimum are captured and sequenced from a whole brain. Assuming one to three infections per neuron (based on the results in Fig. 2), we estimate that at least 1.67 million neurons are screened per brain. Notably, this number can be dramatically boosted by increasing the number of mice for a screen at minimal additional cost. For example, the M3 library-injected cohort of 19 mice represents screening of at least 30 million neurons.

### Estimating the number of mice required for screens

**Fig. 6:**
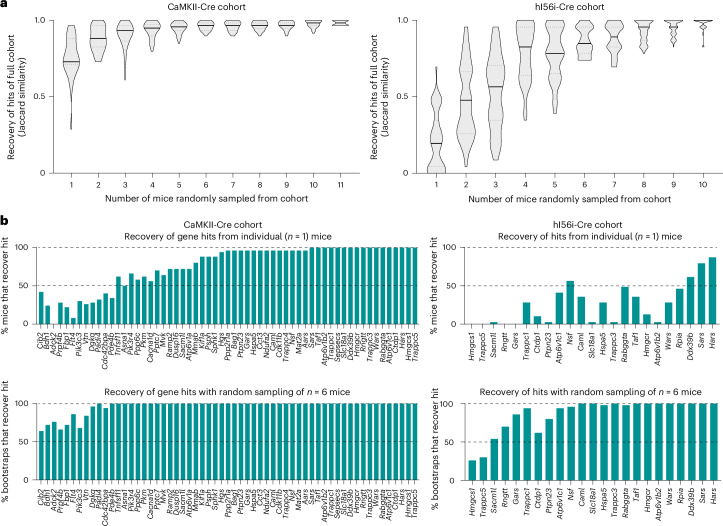
Bootstrapping analysis estimates the number of mice required for different in vivo screening conditions. **a**, Increasing numbers of mice were randomly sampled and bootstrapped from the CaMKII-Cre (left) and hI56i-Cre (right) cohorts to assess how frequently the hits from a bootstrap overlap with the hits identified from their whole respective cohort. Hits are defined by an FDR < 0.1. **b**, Top, the percentage of individual mice that recover each of the hits defined from analyzing the whole cohort. Bottom, percentage of bootstraps when randomly sampling six mice of the cohort that recover each of the hits of the whole cohort. The left half is the CaMKII-Cre cohort and the right half is the hI56i-Cre cohort.

To investigate the impact of cohort size on hit recovery under different Cre conditions, we performed a bootstrap analysis on the CaMKII-Cre screen cohort (from Fig. 4c) and hi56i-Cre screen cohort (from Fig. 5c), both of which were injected with the same preparation and concentration of PHP.eB::pAP215-M1 library. Briefly, we randomly subsampled mice without replacement at incrementing cohort sizes 50 times for each cohort. We measured the extent to which the hits from each subsample overlapped with those defined by the whole cohort (that is, *n* = 12 CaMKII-Cre mice or *n* = 11 hI56i-Cre mice; Fig. 6a).

In the CaMKII-Cre screen, the complete set of hits was recovered with fewer than the complete set of mice; 90% of the hits were recovered with just three mice (Fig. 6a). When examining each of the 61 hits identified from the whole CaMKII-Cre cohort, most genes were called hits even in individual mice. (Fig. 6b, top). When bootstrapping by randomly sampling six mice from the cohort, nearly all hit genes were recovered in most bootstraps. (Fig. 6b, bottom).

In contrast, the hI56i-Cre screen cohort overall showed fewer hits, further supporting that targeting a neuronal subpopulation reduces the power of the screen (Fig. 6a). While few hits were robustly recovered in individual mice (Fig. 6c, top), there was a stronger benefit of additional mice, since most hits were recovered robustly when bootstrapping on six mice (Fig. 6c, bottom).

Together, these findings confirm that relatively few mice are required when screening large neuronal populations, whereas more mice are needed to effectively power screens when screening neuronal subpopulations. In future applications, this bootstrapping approach can be implemented to empirically establish whether an in vivo screen is powered for the given neuronal population and library size.

### Focused screening for essential chaperone genes in vivo

**Fig. 7:**
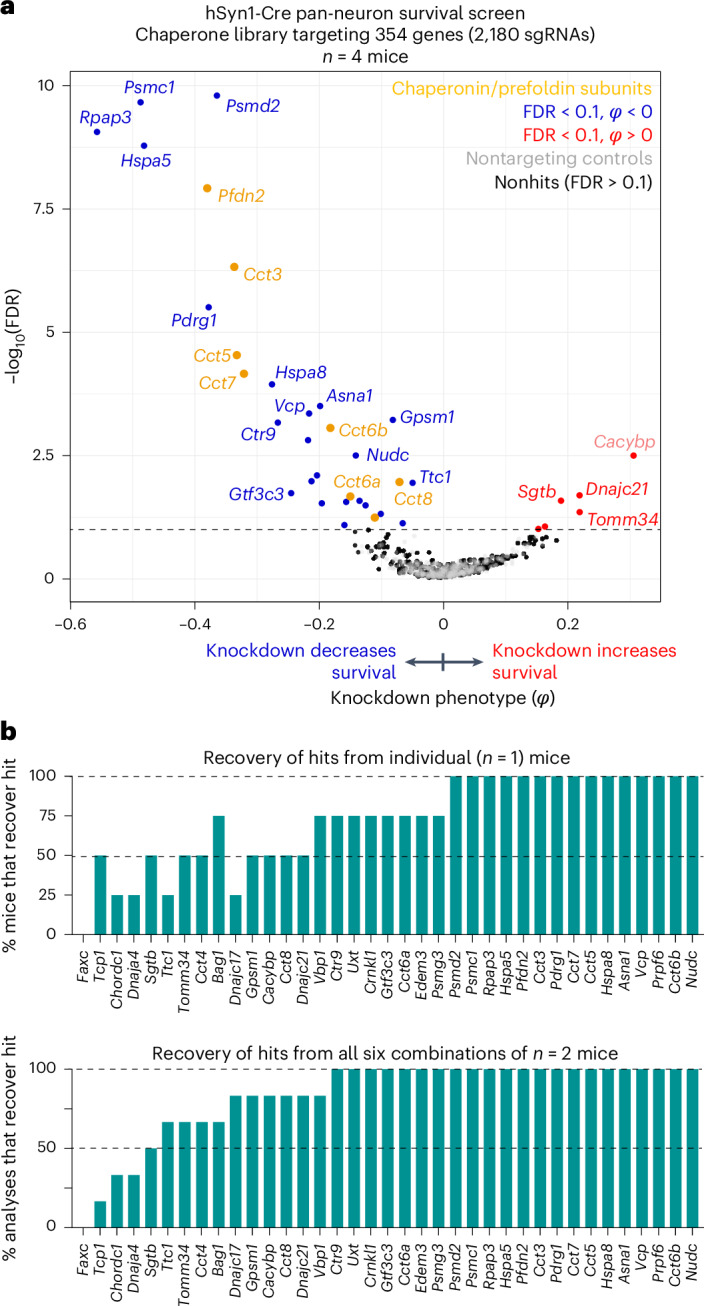
CRISPRi screening for neuron-essential chaperones using a smaller sgRNA library. **a**, Knockdown phenotypes of 354 chaperone genes averaged across four LSL-CRISPRi mice at 6 weeks after neonatal ICV injection of PHP.eB::hSyn1-Cre and a PHP.eB-packaged sgRNA library targeting chaperones (354 genes, 2,180 sgRNAs, of which 350 are nontargeting controls). *Cacybp* is labeled in light pink to reflect that its phenotype was driven by only one sgRNA. **b**, Top, the percentage of individual mice that recover each of the hits defined from analyzing the whole cohort. Bottom, percentage of analyses for all six combinations of two of the four mice that recover each of the hits of the whole cohort.

To test the impact of using a smaller sgRNA library on screening performance by CrAAVe-seq, we designed a library of 2,172 sgRNAs targeting 354 known molecular chaperone and cochaperone genes and cloned it into the pAP215 vector (pAP215-chap library). Given the importance of protein homeostasis in neurons, this would also further establish the chaperones that are critical for neuronal survival. We co-injected PHP.eB::pAP215-Chap library and PHP.eB::hSyn1-Cre into a cohort of four neonatal LSL-CRISPRi mice, followed by CrAAVe-seq at 6 weeks. The screen revealed 32 hit genes, 26 of which had a negative knockdown phenotype, indicating them to be neuron-essential chaperones (Fig. 7a). The hits included several genes that were identified from the M1 and M3 library screens in Fig. 3 (for example, *Hspa5* and subunits of the chaperonin complex). Notably, contrasting the M3 library screen, the chaperonin subunits were even more strongly represented and exhibited a lower FDR, supporting that a reduced library size increased the power of this screen. The high power and sensitivity of this screen, with a focused library, are further corroborated by the finding that more than half of the 32 hits could be captured from one mouse, and this number substantially increases when using two mice (Fig. 7b).

### Validating *Hspa5* and *Rabggta* as essential neuronal genes

We selected two top hits from our in vivo screens, *Hspa5* and *Rabggta*, for individual validation to establish that screening for sgRNA depletion from recovered episomes reflects capture of neuron-essential genes. *Hspa5* and *Rabggta* were also consistently among the strongest hits in individual mice, highlighting two distinct biological pathways. *Hspa5* encodes an ER-associated Hsp70 chaperone, helping facilitate the folding of newly synthesized proteins. Prior work has shown that knockdown or knockout of *Hspa5* in select neuronal populations in vivo leads to their apoptotic cell death^28,29^. *Rabggta* encodes a geranylgeranyl transferase that regulates GTPase activity of Rab proteins, which are involved in intracellular vesicle trafficking. The impact of knocking down these genes broadly in neurons had not been previously examined.

**Fig. 8:**
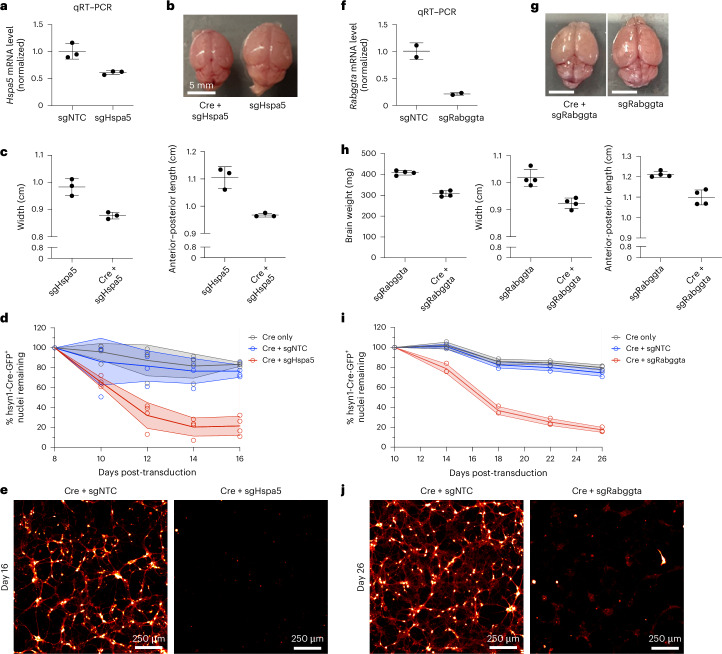
Validation of *Hspa5* and *Rabggta* as neuron-essential genes in mouse neurons. **a**, Primary neurons cultured from constitutive CRISPRi mice were transduced with PHP.eB::pAP215 targeting *Hspa5* (sgHspa5) or nontargeting control (sgNTC). *Hspa5* mRNA levels were assayed by qRT–PCR and normalized to the sgNTC control (mean ± s.d., *n* = 3 technical replicates). **b**, Representative brains of LSL-CRISPRi mice 16 days after neonatal ICV injection of PHP.eB::pAP215-sgHspa5 with or without co-injection with PHP.eB::hSyn1-Cre (Cre). Scale bar, 5 mm. **c**, Quantification of brain width and length (mean ± s.d., *n* = 3 independent mice) from **b**. Gross motor phenotypes for these mice are shown in Supplementary Videos 1 and 2. **d**, Primary neurons cultured from LSL-CRISPRi mice following transduction with PHP.eB::hSyn1-Cre and PHP.eB::pAP215-sgHspa5 or sgNTC (mean ± s.d., *n* = 4 wells). Survival was determined by counting GFP^+^ nuclei over time and normalized to peak fluorescence, which occurred at day 8. **e**, Representative image of primary neuronal cultures from **d** at 16 days post-transduction. Cells were cotransduced with constitutively expressed cytosolic mScarlet (PHP.eB::CAG-mScarlet), which reveals fine neuronal processes (displayed with a red to white lookup table). Scale bar, 250 µm. **f**–**j**, Identical experimental designs to **a**–**e**, except using PHP.eB::pAP215-sgRabggta instead of sgHspa5, with *Rabggta* mRNA levels in primary neurons (**f**), brain appearance at 28 days after injection; scale bars 5 mm (**g**), brain measurements (**h**), neuronal survival quantified across *n* = 3 wells (**i**) and appearance of neurons at 26 days post-transduction; scale bars 250 µm (**j**). Peak fluorescence in cultures quantified in **i** occurred at day 10 and were normalized to that timepoint.

In primary neurons cultured from mice with constitutive CRISPRi machinery, we confirmed that sgRNAs targeting *Hspa5* (sgHspa5) and *Rabggta* (sgRabggta) suppress expression of their respective endogenous transcripts (Fig. 8a,f). Furthermore, injection of PHP.eB::pAP215-sgHspa5 and PHP.eB::hSyn1-Cre into neonatal LSL-CRISPRi mice led to a severe motor phenotype after approximately 2 weeks in mice, but not the sgRNA alone (Supplementary Videos 1 and 2), requiring prompt killing. The brains from mice co-injected with sgHspa5 and hSyn1-Cre were markedly smaller in size relative to littermates with sgHspa5 alone (Fig. 8b,c). In primary neurons cultured from LSL-CRISPRi mice, AAVs delivering sgHspa5 led to marked Cre-dependent neuronal death within 2 weeks of expression (Fig. 8d,e). Similarly, neonatal LSL-CRISPRi mice co-injected with sgRabggta and hSyn1-Cre at 24 days after injection showed a severe motor phenotype, and the brains of these mice were smaller and weighed less in comparison to littermates injected with sgRabggta alone (Fig. 8g,h). LSL-CRISPRi primary neurons transduced with sgRabggta showed Cre-dependent neuronal death, beginning at ~14 days after transduction, with nearly complete death observed at 26 days (Fig. 8i,j). These findings confirm that in vivo CRISPR screening using CrAAVe-seq identifies essential genes that validate even when sgRNAs are introduced into mature primary neurons postnatally.

## Discussion

Here we establish CrAAVe-seq, a platform for cell-type-specific CRISPR-based screening in vivo in mouse tissues with very high scalability due to the Cre-dependent amplification of sgRNA sequences from AAV-derived episomes. Our pan-neuronal CRISPRi screen targeting over one quarter of the protein-coding genes in the mouse genome uncovered neuron-essential genes in the brain with high reproducibility of top hits between individual mice. Additional discussion on select hits, limitations, and additional considerations is provided as Supplementary Note.

This platform can be immediately applied to existing neurodegeneration mouse models to systematically screen for modifiers of neuronal vulnerability in large brain regions, such as the cortex, or in focused populations, such as Purkinje neurons, where the abundance of granule cells might mask biological hits. Moreover, the persistent expression of AAV episomes in nondividing cells^30^ makes CrAAVe-seq well suited for aging studies.

While our screen primarily identified gene perturbations that reduce neuronal survival, future studies in neurodegenerative models could identify perturbations that rescue neuronal survival, revealing potential therapeutic targets. Beyond neurodegeneration, we conceive possible applications to study genetic factors that modify cell survival or growth/proliferation after traumatic brain injury and stroke. Furthermore, CrAAVe-Seq will be useful for screening specific subpopulations of cells throughout the body, leveraging the growing toolkit of AAV capsids and Cre drivers.

## Methods

### Animals

All mice were maintained according to the National Institutes of Health guidelines and all procedures used in this study were approved by the University of California, San Francisco Institutional Animal Care and Use Committee. Mice were housed under a 12-h light/12-h dark cycle at 22–25 °C and 50–60% humidity, with food and water provided ad libitum. Mice were randomly assigned to the experimental groups at the time of injection, and both male and female mice were used. In accordance with approved protocol, mice were monitored postinjection, and if signs of distress appeared, they were documented and killed promptly. The mice used in this study are LSL-dCas9-KRAB (LSL-CRISPRi) mice (B6;129S6-*Gt(ROSA)26Sor*^tm2(CAG-cas9*/ZNF10*)Gers^/J, RRID: IMSR_JAX:033066)^17^ and dCas9-KRAB mice (B6.Cg-*Igs2*^*tm1(CAG-mCherry*,*-cas9*/ZNF10*)Mtm^/J, RRID: IMSR_JAX:030000). A summary of the individual mice used for CRISPR screening and select in vivo experiments is provided in Supplementary Table 1.

### Plasmids

The screening vector pAP215 is shown in Fig. 1a (a fully annotated map is available on Addgene plasmid 217635). Details on cloning pAP215 are provided in Supplementary Protocols. Additional plasmids in this study include pENN.AAV.hSyn.HI.eGFP-Cre.WPRE.SV40 (Addgene, 105540; a gift from J. M. Wilson, University of Pennsylvania), pENN.AAV.CamKII.HI.GFP-Cre.WPRE.SV40 (Addgene, 105551; a gift from J. M. Wilson), CN1851-rAAV-hI56i-minBglobin-iCre-4X2C-WPRE3-BGHpA (Addgene, 164450; a gift from J. Ting, The Allen Institute for Brain Science; ref. ^27^) and pAAV-FLEX-GFP (Addgene plasmid 28304; a gift from E. Boyden, Massachusetts Institute of Technology). The NLS-mScarlet and NLS-mNeonGreen AAV plasmids were generated by restriction cloning, replacing the GFP sequence in plasmid CAG-NLS-GFP (Addgene, 104061; a gift from V. Gradinaru, California Institute of Technology)^18^ and replacing the NLS sequence with one from the pMK1334 plasmid^1^.

### sgRNA cloning

We transferred the sgRNA sequences from our pooled mCRISPRi-v2 sgRNAs, subpools M1-top 5 (targeting kinases, phosphatases and drug targets) and M3-top 5 (targeting the proteostasis network)^22^ into the pAP215 plasmid backbone to create the pAP215-M1 and pAP215-M3 sgRNA libraries, respectively. The mouse chaperone targeting library was designed by selecting the mouse orthologs of a human chaperone targeting library that we previously developed^31^ and included 350 nontargeting control sgRNAs. Oligonucleotide pools were synthesized by Agilent, amplified by PCR, and cloned into the pAP215 backbone. Steps for library cloning are detailed in Supplementary Protocols.

Individual sgRNAs were cloned into the pAP215 backbone, digested with BstXI and Bpu1102I using annealed oligonucleotides (Integrated DNA Technologies) with compatible overhangs. The protospacer sequences for the specific sgRNAs used in this study include sgCreb1 (GGCTGCGGCTCCTCAGTCGG), sgHspa5 (GAACACTGACCTGGACACTT’), sgRabggta (GCGGCGAACTCACCTGCTCA) and a nontargeting control (sgNTC; GGATGCATAGATGAACGGATG).

### AAV packaging, purification and titering

To generate AAV for in vivo injections, two 15-cm dishes were each seeded with 1.5 × 10^7^ HEK293T cells (ATCC, CRL-3216) in 25 ml DMEM complete medium—DMEM (Gibco, 11965-092) supplemented with 10% FBS (VWR, 89510; lot, 184B19), 1% penicillin–streptomycin (Gibco, 15140122) and 1% GlutaMAX (Gibco, 35050061). The next day, 20 µg of pAdDeltaF6 (Addgene, 112867; a gift from J. M. Wilson), 7 µg of library plasmid, 7 µg of pUCmini-iCAP-PHP.eB (Addgene, 103005; a gift from V. Gradinaru)^18^ and 75 µl of 1 mg ml^−1^ polyethenylamine (PEI; Linear, MW 25,000, Polysciences, 23966) were diluted into 4 ml of Opti-MEM (Gibco, 31985062), gently mixed, and incubated at room temperature for 10 min. The PEI/DNA transfection complex was then pipetted dropwise onto the HEK293T cells. After 24 h, the media was replaced with 27 ml of fresh Opti-MEM.

Seventy-two hours after transfection, AAV precipitation was performed as previously described^32^, with modifications. Cold 5× AAV precipitation solution (40% polyethylene glycol (Sigma-Aldrich, 89510) and 2.5 M NaCl) was prepared. The cells and media were triturated and collected (~30 ml) into a 50 ml conical tube, followed by the addition of 3 ml chloroform and vortexing for approximately 30 s. The homogenate was centrifuged at 3,000*g* for 5 min at room temperature, and the aqueous (top) phase was transferred to a new 50 ml conical tube and 5× AAV precipitation solution was added to a final 1× concentration, followed by incubation on ice for at least 1 h. The solution was centrifuged at 3,000*g* for 30 min at 4 °C. The supernatant was completely removed and the viral pellet was resuspended in 1 ml of 50 mM HEPES and 3 mM MgCl_2_, and incubated with 1 µl DNase I (New England Biolabs, M0303S) and 10 µl RNase A (Thermo Fisher Scientific, EN0531) at 37 °C for 15 min. An equal volume of chloroform was added, followed by vortexing for 15 s, and centrifuged at 16,000*g* for 5 min at room temperature; this step was repeated once. Using 400 µl at a time, the aqueous phase was passed through a 0.5-ml Amicon Ultra Centrifugal Filter with a 100 kDa cutoff (Millipore, UFC510024) via 3 min of centrifugation at 14,000*g*, followed by buffer exchange twice with 1× DPBS. Titering was performed by quantitative RT–PCR as previously described^33^ using primers (Integrated DNA Technologies) listed in Supplementary Table 4. This method of AAV production is available as a companion protocol in ref. ^34^.

To prepare AAV for testing in primary neuronal cultures (for longitudinal imaging and qRT–PCR), HEK293T cells were seeded into a 6-well plate containing 1.5 ml of complete DMEM medium. The cells were transfected with 1 µg of pAdDeltaF6, 350 ng of pUCmini-iCAP-PHP.eB and 350 ng of the AAV transgene using PEI as described above. Approximately 48 h after transfection, the cells and media were collected in a 2-ml microfuge tube, 200 µl of chloroform was added to each tube, vortexed for 15 s, and centrifuged at 16,000*g* for 5 min at room temperature. The aqueous (top) phase was transferred to a new tube and AAV precipitation solution was added to 1× dilution, and incubated on ice for at least 1 h. The precipitated AAV was centrifuged at 16,000*g* for 15 min at 4 °C. The supernatant was removed, the pellet was resuspended in 100 µl of 1× PBS and centrifuged again at 16,000*g* for 1 min to remove excess debris. The supernatant (purified virus) was transferred to a new microfuge tube. Ten microlitres of purified virus were used per well in primary neuronal cultures in a 24-well plate.

### ICV injection

ICV injections were performed as previously described, with minor modifications^35^. Briefly, neonatal mice were placed on a gauze-covered frozen cold pack and monitored for complete cryoanesthesia. The scalp was gently cleaned with an alcohol swab. AAVs were diluted in 1× PBS with 0.1% trypan blue into a 2 µl final volume per mouse and loaded into a 10-µl syringe (Hamilton, 1701). The syringe was equipped with a 33-gauge beveled needle (Hamilton, 7803-05; 0.5 inches in length). The needle was inserted through the skull at a point two-fifths of the distance from the lambda suture to the eye, to a depth of 3 mm, to target the left lateral ventricle. Following a one-time unilateral injection, the neonate was placed on a warming pad for recovery and returned to the parent cage. The number of viral particles injected in each mouse is listed in Supplementary Table 1.

### sgRNA recovery and sequencing for CrAAVe-seq

Animals were killed using CO_2_, and their whole brains were removed and stored at −80 °C. The sex of the mice was recorded before killing.

#### Initial protocol for sgRNA recovery

This protocol for episome recovery was used in the following figures—Fig. 1d, M1 library screen in Fig. 3; the hSyn1-Cre screen in Fig. 5d, the hSyn1-Cre versus no Cre screens in Extended Data Fig. 6 and the screens in Extended Data Fig. 7. Each brain was placed in a PYREX 7 ml Dounce Homogenizer (Corning, 7722-7) with 2 ml of TRIzol (Invitrogen, 15596026) and thoroughly homogenized using the a pestle (0.0045 nominal clearance) for ten or more strokes. A volume of 0.4 ml chloroform was added, vigorously shaken for 30 s and centrifuged at 12,000*g* for 15 min at 4 °C. The aqueous phase (top) was collected and nucleic acids precipitated using 1 ml isopropanol, incubated on ice for 10 min and centrifuged at 12,000*g* for 10 min at 4 °C. The supernatant was discarded and the pellet was washed in 2 ml of 75% ethanol in DNase/RNase-free water and spun down at 7,500*g* for 5 min. The supernatant was then removed and the pellet was allowed to air dry for 10 min, and then resuspended in 100 µl of DNase/RNase-free water and incubated with 1 µl of RNase A (Thermo Fisher Scientific, EN0531) at 37 °C overnight. The sample was then column purified using Zymo DNA Clean and Concentrator-25 kit (Zymo Research, D4033) and eluted in 50 µl of DNase/RNase-free water to yield recovered viral DNA. The remaining RNAse-treated samples were considered recovered episomes for use in PCR, as described below.

#### Optimized protocol for sgRNA recovery

An optimized protocol for episomal sgRNA recovery was used in the following figures—the M3 library screen in Fig. 3, the CaMKII-Cre screens in Fig. 4, the hi56i-Cre screens in Fig. 5 and for the digital PCR experiments in Fig. 5. All steps in this protocol are the same as the above initial protocol except for two modifications. First, each brain was homogenized in 4 ml of TriZOL, phase separated using 0.4 ml chloroform and the aqueous phase precipitated with 2 ml isopropanol, before resuspending in 100 µl of DNase/RNase-free water. Second, following overnight RNase A treatment as above, the sample was directly transferred to −20 °C without column purification. The optimized protocol is available as a companion protocol in ref. ^14^.

#### PCR amplification of sgRNAs and sequencing

The PCR was performed using Q5 High-Fidelity 2× master mix (New England Biolabs, M0492L). Each reaction contained 100 µl of recovered episomes, 110 µl of Q5 2× master mix and 5.5 µl of each primer. For amplification of the AAV sgRNA libraries, the purified AAV was diluted tenfold into H_2_O, and 1 µl of the diluted AAV was used as a template in a 100 µl PCR reaction. The reaction was distributed into PCR tubes at the maximum volume allowed by the PCR equipment. The following PCR cycling conditions were used: 98 °C for 30 s; followed by 23 cycles of 98 °C for 30 s, 60 °C for 15 s and 72 °C for 15 s; with a final extension at 72 °C for 10 min.

Each 100 µl PCR reaction was purified using 1.1× SPRI beads (SPRIselect Beckman Coulter, B23317) and resuspended in 25 µl elution buffer (Machery Nagel, 740306). The purified products were pooled and sequenced on an Illumina HiSeq 4000 at the UCSF Center for Advanced Technologies or on an Illumina NextSeq 2000 and demultiplexed with Illumina Dragen BCL Convert. The amplification primers (with adapters) and custom sequencing primers (Integrated DNA Technologies) are listed in Supplementary Table 4. See Supplementary Information for details of digital PCR experiments.

### Mouse cortical neuron primary cultures and immunocytochemistry

Neonates were briefly sanitized with 70% EtOH and decapitated using sharp scissors, and the brains were removed and placed into cold HBSS (Gibco, 14175095). The meninges were removed under a dissecting microscope, and the cortices were transferred to a 15-ml conical tube containing 10 ml of 0.25% trypsin–ethylenediaminetetraacetic acid (EDTA) (Gibco, 25200056) and incubated at 37 °C for 30 min. The trypsin was removed and the brains were gently rinsed twice in 5 ml of DMEM complete media, followed by trituration of brains in 5 ml of DMEM complete media filtered through a 40 µm nylon cell strainer (Corning, 352340), and diluted into DMEM complete media in a volume as needed for plating. An equivalent of one brain was plated across each BioCoat poly-d-lysine 24-well TC-treated plate (Corning, 356414). The following day, day in vitro 1 (DIV1), the DMEM complete media was replaced with neuronal growth media composed of Neurobasal-A Medium (Gibco, 10888022), 1× B-27 Supplement minus vitamin A (Gibco, 12587010), GlutaMAX Supplement (Gibco, 35050079) and 1% penicillin–streptomycin (Gibco, 15140122). On DIV2, the cultures were further supplemented with cytarabine (AraC) to a final concentration of 200 µM (Thermo Scientific Chemicals, 449561000). The primary neuronal cultures were transduced with AAV on DIV4 and imaged starting 4 days after transduction. See Supplementary Information for details on RNA isolation, qRT–PCR, live-cell imaging and quantification of cell death.

### Mouse brain immunofluorescence staining

Whole brains were removed and fixed overnight at 4 °C in 4% paraformaldehyde (Electron Microscopy Sciences, 15710) diluted in 1× PBS. The following day, the fixative was replaced with 30% sucrose dissolved in 1× PBS for at least 48 h. Fixed brains were blotting dry, cut down the midline with a razor blade, and mounted into a cryomold (Epredia, 2219) using OCT compound (Sakura Finetek, 4583). To snap freeze, cryomolds were partially submerged in a pool of 2-propanol cooled by a bed of dry ice. Brains were sectioned in the sagittal plane at 40 µm on a cryostat (Leica, CM1950) with a 34° MX35 Premier+ blade (Epredia, 3052835). The resulting brain sections were stored free-floating in 1× PBS + 0.05% NaN_3_ at 4 °C. When ready for staining, representative brain sections were washed thrice in 1× PBS and incubated in a 24-well plate at room temperature for 1 h in blocking buffer—10% goat serum (Gibco, 16210064), 1% BSA (Sigma-Aldrich, A7906) and 0.3% Triton X-100 (Sigma-Aldrich, T8787) diluted in 1× PBS. The brain sections were incubated overnight in primary antibodies diluted in blocking buffer at 4 °C on a gentle shaker. The sections were washed three times in 1× PBS, then incubated in secondary antibodies for 2 h at room temperature in the dark on a gentle shaker. Sections were washed thrice in 1× PBS and moved to charged glass microscope slides (Thermo Fisher Scientific, 12-55015). After PBS was removed, Fluoromount-G with DAPI mountant (Invitrogen, 00-4959-52) was added, and a No. 1.5 coverslip (Globe Scientific, 1415-15) was placed on top. Slides were dried at room temperature in the dark overnight and sealed with nail polish. For experiments without DAPI, ProLong Gold Antifade mountant (Invitrogen, P10144) was used instead. For experiments with Hoechst instead of DAPI, sections were lastly incubated for 15 min in Hoechst 33342 (BD Pharmingen, 561908) diluted 2 µg ml^−1^ in 1× PBS, then washed thrice in 1× PBS before mounting using ProLong Gold mountant.

The following primary antibodies were used: rabbit anti-CREB (1:1,000 dilution; clone, 48H2; Cell Signaling Technologies, 9197), rabbit anti-SOX9 (1:2,000 dilution; polyclonal; EMD Millipore, AB5535), guinea pig anti-NeuN (1:500 dilution; polyclonal; Synaptic Systems, 266004), alpaca FluoTag-Q anti-TagFP nanobody (1:500 dilution; clone, 1H7; Alexa647 pre-conjugated; NanoTag Biotechnologies, N0501-AF647-L), which reacts to mTagBFP2 but not eGFP. The following secondary antibodies were used: goat antirabbit IgG Alexa Fluor 488 (1:1,000 dilution; Invitrogen, A-11034), goat antirabbit IgG Alexa Fluor 568 (1:1,000 dilution; Invitrogen, A-11011), goat antirabbit IgG Alexa Fluor 647 (1:1,000 dilution; Invitrogen, A-21245), goat antiguinea pig IgG Alexa Fluor 488 (1:1,000 dilution; Invitrogen, A-11073) and goat antiguinea pig IgG Alexa Fluor 647 (1:1,000 dilution; Invitrogen, A-21450). All secondary antibodies were highly cross-absorbed. See Supplementary Information for imaging parameters and quantification.

### CRISPR screen analysis

Computational analysis of the screen data was performed using a newly developed bioinformatics pipeline, which is publicly available (Code availability). Raw sequencing results were mapped to the M1 protospacer library using ‘sgcount’^36^. Briefly, ‘sgcount’ is a tool to match protospacers against a reference protospacer library with exact pattern matching. The resulting count matrices, containing guide and gene information along with count data for each sample, were used as input for subsequent analyses.

The ‘crispr_screen’^37^ was used to perform differential sgRNA abundance analysis and gene score aggregation analysis. The ‘crispr_screen‘ is a reproduction of the original MAGeCK analysis, but performs differential sgRNA analysis using a negative binomial as originally described in the study, and not a truncated normal distribution as used in the current MAGeCK implementation.

In brief, sgRNA abundances are median normalized across samples, then a weighted linear regression (weighted ordinary least squares) is used to fit the log variance to the log mean of the control samples (representing sgRNA abundances in the AAV library). The fit variance and mean are then used to parameterize negative binomial distributions for each sgRNA and a survival function or cumulative distribution function is used to calculate a *P* value for sgRNA underabundance and overabundance. We excluded any sgRNAs that were represented with fewer than 100 reads across the control AAV samples.

To calculate a gene-level aggregated metric across sgRNAs of the same gene group, we established a new algorithm, geometric *P* value aggregation or geopagg. We performed the following operations on the underabundance and overabundance *P* values in parallel. First, the differential abundance *P* values for sgRNAs were corrected for multiple hypothesis correction using the Benjamini–Hochberg correction procedure to calculate an FDR for each sgRNA. Next, FDRs for sgRNAs belonging to the same gene grouping were collected and sorted in ascending order. We then calculated a weighted geometric mean FDR (${q}_{i}$) for each gene (${i\in N}$) across the FDRs (${x}_{j}$) for sgRNAs within the gene group (${j\in M}$).

$${q}_{i}\,={\mathrm{exp}}\left(\mathop{\sum }\limits_{j}^{{M}_{i}}\frac{{w}_{j}{\mathrm{ln}}{x}_{j}}{\mathop{\sum }\limits_{j}^{{M}_{j}}{w}_{j}}\right)$$

We calculated a weighted geometric mean to down-weight the relative impact of the first sgRNA within the group using a ‘Drop-First’ weighting strategy. The first sgRNA (or top-performing sgRNA) is down-weighted because we generally aim to capture genes with multiple high-performing sgRNAs. The weights for each gene grouping ($${M}_{i})$$ are defined as follows:

$${w}_{j}\,=\,\left\{\begin{array}{c}0.5\;{\rm{if}}\;{x}_{j}={\text{min}}\left[{x}_{k}\,\forall \,k\in {M}_{i}\right]\\ 1.0\;{\text{if}}\;{x}_{j}\ne {\text{min}}\left[{x}_{k}\,\forall \,k\in {M}_{i}\right]\end{array}\right.$$

We also performed an aggregation of the log_2_-fold-changes in abundance (a gene’s phenotype score) of each sgRNA (${\varphi }_{j}$) within the gene group with an arithmetic mean:

$${\varphi }_{i}=\frac{1}{{M}_{i}}\left(\mathop{\sum }\limits_{j}^{{M}_{i}}{\varphi }_{j}\right)$$

We then created random groupings of sgNTC, which we denote as the amalgam gene set ($A$), to match the gene membership distribution of the input sgRNA library. This was performed by determining the membership size (number of sgRNAs) of each gene (${M}_{j}$) and sampling an equal amount of sgRNAs without replacement from the nontargeting controls. We next performed an identical calculation as above for each of the newly created amalgam genes.

We then calculated a ‘gene score’ for each gene and each amalgam gene within the dataset using the calculated weighted geometric mean of the FDR values ${q}_{i}$) and their phenotype score (${\varphi }_{i}$).

$${{\rm{\gamma }}}_{i}=\left({\varphi }_{i}\right)\left(-{\log }_{10}{q}_{i}\right)$$

We next sort the gene scores (${\gamma }_{i}$) in an ascending order or a descending order for the underabundant and overabundant tests, respectively.

Finally, we calculated an empirical FDR (*δ*_*i*_) by stepping through the weighted geometric mean (*q*) arrays and determining for each rank (*i*) how many amalgam genes (${g}_{i}\in A$) are preceding it. Because the true empirical FDR will be zero for all genes preceding the first amalgam gene, we provided a nonzero score by constraining the reported FDR to be the maximum of the empirical FDR and the weighted geometric mean of that gene.

$${\delta }_{i}\,={\mathrm{max}}\left(\frac{{c}_{i}}{i}\,|\,{q}_{i}\right)$$

$${c}_{i}\,=\,\mathop{\sum }\limits_{1}^{i}\left\{\begin{array}{c}1\;\text{if}{\;g}_{i}\in A\\ 0\;\text{if}{\;g}_{i}\notin A\end{array}\right.$$

This empirical false discovery is further constrained for explicit monotonicity by requiring the current score to be greater than or equal to the previous one.

$${\delta }_{i}\,={\mathrm{max}}\left({\delta }_{i}\,|\,{\delta }_{i-1}\right)\,\forall \,i\, > \,1$$

The geopagg algorithm is performed for the sgRNA underabundant and overabundant *P* values in parallel and the final scores for each gene are reported as the most significant of the two tests.

A total of 151 genes in the M1 library and 129 genes in the M3 library are targeted at two different transcriptional start sites by different sets of sgRNAs. These sets were evaluated independently, labeled as P1 and P2 (for example, GeneA_P1 and GeneA_P2). In cases where only one set is significant and labeled on a heatmap or volcano plot, the P1 or P2 label is not shown, but this information is included in Supplementary Tables 2 and 3.

Details on the bootstrapping analysis are provided in Supplementary Information.

### Statistics and reproducibility

No statistical methods were used to predetermine sample sizes, but our sample sizes are similar to those reported in previous publications, as cited in Main. Numbers of replicates and number of mice used are stated in figures or figure legends (Figs. 2–5, 7 and 8 and Extended Data Figs. 4 and 7–9). In all figures that show a representative fluorescence micrograph (Figs. 1, 2, 4, 5 and 8 and Extended Data Figs. 2–5), the experiments were repeated at least once to verify similar findings. No repeat measurements were made on the same samples. Data were assumed to be normally distributed except for instances within the ‘crispr_screen’ pipeline where geopagg uses a negative binomial distribution to calculate *P* value. The ‘crispr_screen’ pipeline was used with FDR < 0.1 and controls for multiple comparisons using the Benjamini–Hochberg correction. For cell culture experiments, randomization was not performed because the treatment groups of cells were derived from the same parent population. Data collection and analysis were not performed in a blinded manner to the conditions of the experiments. No animals or data points were excluded from the relevant analyses. Major findings were validated using independent samples and orthogonal approaches.

### Reporting summary

Further information on research design is available in the Nature Portfolio Reporting Summary linked to this article.

## Online content

Any methods, additional references, Nature Portfolio reporting summaries, source data, extended data, supplementary information, acknowledgements, peer review information; details of author contributions and competing interests; and statements of data and code availability are available at 10.1038/s41593-025-02043-9.

## Supplementary information

Supplementary Information Supplementary Note (Supplementary Discussion and Supplementary Protocols).

Reporting Summary

Supplementary Tables 1–5 Supplementary Tables 1–5.

Supplementary Video 1 Gross motor phenotypes in sgHspa5 + hSyn1-Cre-injected mice. LSL-CRISPRi neonates were co-injected by ICV with AAV containing sgHspa5 (in pAP215) and AAV containing hSyn1-Cre. At 16 days postinjection, mice displayed gross motor phenotypes, as recorded in the video. After video capture, mice were immediately killed as per IACUC protocol. Mice were littermates to those shown in Supplementary Video 2 and injected and recorded at the same timepoint.

Supplementary Video 2 Lack of gross motor phenotypes in sgHspa5 only injected mice. LSL-CRISPRi neonates were co-injected by ICV with AAV containing sgHspa5 (in pAP215) and an equivalent volume of phosphate-buffered saline. At 16 days postinjection, mice displayed normal motor phenotypes, as recorded in the video. Mice were littermates to those shown in Supplementary Video 1 and injected and recorded at the same timepoint.

## Source data

Source Data Fig. 1 Unprocessed gel from Fig. 1d.

Source Data Fig. 5 Unprocessed gel from Fig. 5e.

## Data Availability

All data are publicly available at the Dryad data repository (10.5061/dryad.0k6djhb9t)^38^. Data from the DepMap database used to generate Extended Data Fig. 6a is publicly accessible (https://depmap.org/portal)^23^. There are no restrictions on data availability. Source data are provided with this paper.

## Code availability

For CRISPR screen analysis, we developed a highly efficient analysis toolkit called ‘sgcount’ for sgRNA mapping and ‘crispr_screen’ for differential gene abundance testing. The sgRNA mapping utility (‘sgcount‘, version 0.1.32) is also available on GitHub (https://github.com/noamteyssier/sgcount) and Zenodo (https://zenodo.org/doi/10.5281/zenodo.12774352)^36^. The differential gene abundance tool (‘crispr_screen’, version 0.2.8) is also available on GitHub (https://github.com/noamteyssier/crispr_screen/) and Zenodo (https://zenodo.org/doi/10.5281/zenodo.12774208)^37^. All bootstrapping analyses were performed using a custom Python package (‘rescreener’, version 0.1.0) available on GitHub (https://github.com/noamteyssier/bootstrap_analysis_invivo_crispr_screen).

The R notebooks for analysis are available at https://kampmannlab.ucsf.edu/article/scripts-vivo-screening-manuscript. The CellProfiler pipelines are available on the Dryad data repository (10.5061/dryad.0k6djhb9t)^38^ and at https://kampmannlab.ucsf.edu/article/scripts-vivo-screening-manuscript.
