# Supplementary material for: CRISPR screening by AAV episome-sequencing (CrAAVe-seq): a scalable cell-type-specific in vivo platform uncovers neuronal essential genes
Source: Nat Neurosci. 2025 Aug 22;28(10):2129–40. doi: 10.1038/s41593-025-02043-9 (PMC12497649; doi:10.1038/s41593-025-02043-9)
Supplement: Supplementary file 1 — Supplementary Note (Supplementary Discussion and Supplementary Protocols). [file 41593_2025_2043_MOESM1_ESM.pdf]

nature neuroscience

Supplementary information https://doi.org/10.1038/s41593-025-02043-9

CRISPR screening by AAV episome-sequencing (CrAAVe-seq): a scalable cell-type-specific in vivo platform uncovers neuronal essential genes

In the format provided by the authors and unedited

CRISPR screening by AAV episome-sequencing (CrAAVe-seq): a scalable cell type-specific *in vivo* platform identifies neuronal essential genes

Supplementary Text

Supplementary Discussion

Scalability of CrAAVe-seq

CRISPR screens performed in cell culture using lentivirus, which integrate their DNA into the host genome, require extraction of genomic DNA before PCR amplification of the integrated sgRNAs. In contrast, recombinant AAV genomes are mostly maintained as circular or concatenated DNA episomes, with a minor fraction integrating into the host genome[39–41]. Consequently, a major potential advantage of AAV is that the viral genomes can be precipitated and concentrated from the aqueous phase of a TRIzol-chloroform extraction as previously demonstrated in the context of AAV capsid screening[12,13], which could vastly improve scalability for a pooled CRISPR screen. For example, if PCR of sgRNAs from genomic DNA was necessary, a whole mouse brain weighing approximately 500 mg is expected to yield up to 1500 µg of genomic DNA. Since PCR reactions have an upper limit for the amount of template DNA (typically 10 µg per 100 µl reaction)[42,43], PCR recovery of sgRNAs from a screening across a single brain would require up to 15,000 µl of PCR reaction volume. This imposes a severe restriction on scalability, as large volumes of PCR reactions become economically and practically infeasible, especially for screening libraries necessitating dozens of mice.

Genes that uniquely modify neuronal survival *in vivo*

Our *in vivo* screening approach identified several genes have been previously recognized as common essential genes by DepMap[23] or in our prior screens in iPSC neurons[2]. Some hits were unique to the *in vivo* screen, and include *Jtb* (involved in cytokinesis), as well as *Snx17 and Snx20,* members of the sorting nexin family. Therefore, our screens already begin to uncover neuronal vulnerability genes in the mouse brain that have not been previously documented. Conversely, some hits were strong essential genes in our screens in mono-cultured human iPSC-derived neurons, such as *SOD1* and genes involved in the exocyst complex (*EXOC3, EXOC7*), possibly because they cause vulnerabilities that are buffered in the context of the brain. Our screens also uncovered a few genes whose knockdown produced a positive phenotype, and include *Fbxl16*, *Gaa*, *Dstn*, and *Kdelc16*. It is unclear whether these gene knockdowns increase resiliency from death or from inducing proliferation of a transduced cell population; they warrant further investigation.

Considerations and limitations for applying and optimizing CrAAVe-seq

When applying CrAAVe-seq to new applications, factors such as sgRNA library size and viral tropism must be considered. Unlike CRISPR screens in cultured cell-based systems, where low MOI ensures that each cell mostly receives only one sgRNA, the complexity of CNS architecture and viral tropism in different brain regions makes controlling global MOI challenging, as each region will have a local MOI. In our studies, the MOI across the brain was generally 1 to 2, with a few areas exceeding 3. Despite this variability, the large screening library and large number of transduced neurons greatly buffers the potential for complex sgRNA interaction within the same cell, ensuring discernible phenotypes even when randomly paired with other sgRNAs. We found that reducing AAV library concentration significantly diminishes screen performance, indicating that transducing a large number of neurons is more critical than maintaining a low MOI. We also observed, through bootstrapping analyses, that a screen on smaller neuronal populations requires a larger number of mice. Future CNS screens must balance library size, target population(s) with

consideration of dissected brain regions, and desired phenotypes, and we recommend using digital PCR and bootstrap analyses in optimizing for *in vivo* screens.

Our Cre-dependent sgRNA recovery strategy enables highly sensitive screens in smaller neuronal subpopulations, providing an avenue for CRISPR screening on molecularly defined cell types that can be accessed through specific Cre-drivers, or brain regions that can be physically dissected. One area for ongoing optimization is increasing sgRNA recovery and coverage across cell populations. Our current proof-of-concept studies rely on co-injecting AAVs for the sgRNA libraries and Cre recombinase, but using transgenic Cre mouse lines could maximize active sgRNA expression without relying on co-infection. Additionally, temporal control over gene perturbation can be achieved by delivering sgRNA libraries later, via intravenous delivery, or by crossing LSL-CRISPRi mice to Cre^{ERT2} lines for tamoxifen-inducible activation. Furthermore, the Lox71/Lox66 system used for unidirectional handle inversion may not be the maximum possible efficiency, so we are developing a FLEX-based AAV sgRNA backbone for more efficient Cre-dependent handle switching. So far, our screens using different Cre drivers showed strong correlation between independent screens, but no clear evidence of genes essential only in specific neuronal populations. This could be due to the genes included in the M1 library, which may not be optimally suited to detect population-specific vulnerabilities. It may also require longer screening durations to identify genes that impart differential susceptibility.

A minor limitation of using non-integrating AAVs is the gradual loss of AAV episomes during cell division, reducing the fraction of cells from which phenotypic data can be collected, particularly in proliferating tissues. Consequently, this approach would not be best suited to screen for phenotypes related to increased cellular proliferation. This is not a major issue for neurons, as they are postmitotic, and this still permits screening for genes that prevent neuronal death in disease models. For other proliferating cell types, integrating a transposase system into the host genome could help maintain sgRNA expression, as demonstrated previously[9,44]. While this would partly negate the advantage of recovering sgRNAs from episomes, the other advantages afforded by AAV, including superior biodistribution and safety profile remain valuable.

Summary and future directions

CrAAVe-seq complements orthogonal *in vivo* CRISPR screening techniques but is especially well suited for probing specific cell populations with far larger sgRNA libraries for unbiased biological discovery. By taking advantage of widespread AAV transduction in the brain, CrAAVe-seq allows screening across millions of neurons per mouse, which was not feasible with prior options, and this can be further scaled by using multiple mice per screen. While CrAAVe-seq does not provide the same cell-type resolution as scRNA-seq-based Perturb-seq, CrAAVe-seq offers a practical and far less expensive approach for large-scale initial screens using broader libraries (>1,000s of sgRNAs vs. ~10s of sgRNAs) and larger targeted cell populations (~millions vs. 10,000s of cells). We are currently in developing a genome-wide library in the pAP215 backbone for future CrAAVe-seq applications. While our current work demonstrates the use of CrAAVe-seq to uncover modifiers of cell survival, the strategy can be expanded to other relevant phenotypes in the future. For example, delivering an AAV library *in utero* could be used to comprehensively profile the genes involved in the migration and differentiation of different cell types in development. As for previous screens in cultured cells, the use of reporters and fluorescent read-out in conjunction with flow cytometry will enable the identification of modifiers of a plethora of cellular phenotypes. Thus, CrAAVe-seq has the potential to accelerate the rate of biological and therapeutic discovery in relevant animal models while minimizing cost and animal use.

Supplementary Protocols

Cloning pAP215 plasmid

pAP215 was generated using the pAAV-U6-sgRNA-CMV-GFP plasmid as the starting backbone (Addgene plasmid # 85451, a gift from Hetian Lei)[36]. We replaced the sgRNA scaffold sequence with one from pMK1334 (Ref. [1]) and the mU6 using a gene block (gBlock, IDT technologies) using a modified mU6 sequence as reported in Addgene plasmid #53187 (Ref. [37]). The CMV-EGFP module was replaced with EF1a-2xmycNLS-tagBFP2 from pMK1334 by Gibson Assembly. The W3 terminator was cloned from Cbh_v5 AAV-saCBE C-terminal (Addgene plasmid # 137183, a gift from David Liu)[38]. The hGH was replaced by the SV40 from pMK1334. The Lox66 and Lox71 sequences and their orientation were copied from the pFrt-invCAG-Luc (Addgene plasmid # 63577, a gift from Ivo Huijbers)[39] and were inserted along with the 175-bp intervening spacer as a gBlock.

Lentivirus packaging, purification, and injection

The pLG15 vector containing a non-targeting control sgRNA was packaged into lentivirus as previously performed[44] by using PEI for transfecting 15 mg of the transfer plasmid and 15 mg of lentiviral packaging plasmids (containing 1:1:1 pRSV, pMDL, pVSV-G) into 1.0×10^7 HEK293T cells cultured in a 15-cm dish in DMEM complete medium. 48 hours after transfection, the virus was precipitated from the media supernatant using Lentivirus Precipitation Solution (Alstem, VC100) and resuspended in 500 µl of PBS, and then further concentrated using the 0.5-ml Amicon Ultra Centrifugal 100 kDa Column. 1.8 µl of virus plus 0.2 µl of 1% Trypan Blue was injected by ICV for each neonatal mouse. Mouse brains were extracted on day 14 and sectioned coronally.

sgRNA library cloning

20 µg of the M1- or M3-CRISPRiv2 sgRNA library was digested with BstXI (Thermo Scientific, FD1024) and Bpu1102I (Thermo Scientific, FD0094). The guide-encoding inserts (84 bp) were resolved on a 4-20% Novex TBE gel (Invitrogen, EC62252BOX) and precipitated with GlycoBlue and sodium acetate. Inserts were washed with ethanol after precipitation and then eluted in DNase- and RNase-free water. 20 µg of the backbone vector, pAP215, was digested in parallel with BstXI and Bpu1102I, resolved on a 1% agarose gel, and purified from the gel (Zymo Research, D4001). The vectors and insert guides were annealed for 16 hrs overnight using T4 ligase (New England Biolabs, M0202L) at a 1:2 molar ratio of vector to insert, and then purified with sodium acetate and ethanol washing. After the final wash, a portion of the ligated library product was transformed into chemically competent *E. coli* (Takara, 636763) and 10 colonies were picked at random to ensure that each colony contained a unique sgRNA sequence. The remainder of the library product was electroporated into Mega-X competent cells (Invitrogen, C640003) and grown overnight, and a portion of the culture was plated to determine if a coverage of at least 250 colonies per guide was achieved, followed by growth of the remainder of the culture in 1 L of LB for 16 hrs and purification of the library using ZymoPURE II Plasmid Gigaprep Kit (Zymo Research, D4204).

RNA isolation and quantitative RT-PCR

Using CRISPRi primary neurons at 11 days after transduction of AAV (sgNTC, sgHspa5, or sgRabggta), RNA was isolated with the Zymo Quick-RNA Microprep Kit (Zymo Research, R1050). Samples were prepared for qPCR in technical replicate in 10 µl reaction volumes using SensiFAST SYBR Lo-ROX 2× Master Mix (Bioline, BIO-94005), custom qPCR primers from Integrated DNA Technologies used at a final concentration of 0.2 µM and cDNA diluted at 1:20 by

volume. qPCR was performed on a Bio-Rad CFX96 Real Time System C1000 Touch Thermocycler running CFX Maestro (version 4.1.24.33.12.19). The following cycles were run (1) 98°C for 3 min; (2) 95°C for 15 s (denaturation); (3) 60°C for 20 s (annealing/extension); (4) repeat steps 2 and 3 for a total of 39 cycles; (5) 95°C for 1 s; (6) ramp 1.92°C s^{-1} from 60°C to 95°C to establish melting curve. Expression fold changes were calculated using the ΔΔCt method, normalizing to housekeeping gene *Gapdh.* RT-qPCR primers are listed in **Supplementary Table 4**.

Digital PCR

Digital PCR (dPCR) was performed to quantify percent of episomal sgRNAs with an inverted Handle sequence collected from mouse brains with or without hSyn1-Cre or hI65i-Cre. Episomal samples from the CrAAVe-seq post-RNase treatment step were diluted 1:50 in water prior to analysis. Two sets of primers were used: one for total sgRNAs (oIR020/oIR021, 132 bp amplicon) and one for sgRNAs with an inverted handle (oIR022/oIR023, 137 bp amplicon) with sequences in Supplemental Table 4. 15 µl dPCR reactions were prepared using 5 µl EvaGreen 3× PCR master mix (Qiagen, 250111), 1 µl of diluted episomal sample, 1.5 µl 10X primer mix (final primer concentration 0.4 µM each), and 7.5 µl nuclease-free water; 13 µl of this reaction was loaded into the microplate. dPCR was performed on a QIAcuity One 5-plex Digital PCR instrument (Qiagen) using 8.5k-partition, 24-well nanoplates (Qiagen, 250011). The thermal cycling conditions consisted of an initial 2 min at 95°C, followed by 40 cycles of 15 sec at 95°C, 15 sec at 60°C, and 15 sec at 72°C, with a final 5 min cooling step at 40°C. Image capture exposure was set to 250 ms. Samples included M1 library + hSyn1-Cre, M1 library only, and M1 library + hI56i-Cre, with four separate mice for each condition, analyzed for both total and inverted products. Analysis was performed on QIAcuity Software Suite (version 2.5.0.1). A common threshold of 75 RFU was set for all samples. Absolute concentration results for inverted sgRNAs were divided by total sgRNAs for each sample and multiplied by 100 to determine percent inverted sgRNAs.

Microscopy, image segmentation, and analysis

Slides containing brain sections were imaged using a Zeiss AxioScan.Z1 with a Zeiss Colibri 7 unit, ×20/0.8 NA objective lens, 5-30 ms exposure, 1×1 binning and 25-100% intensity using 425-nm, 495-nm, 570-nm and 655-nm lasers, running ZEN version 2.6 software. The images were imported into QuPath (version 0.4.2) for analysis[45]. The raw CZI files are available on Dryad repository (see Data Availability).

To identify overlap between BFP, NeuN, and SOX9, a representative region of the cortex was outlined and the nuclei were segmented on the DAPI channel using the ‘Cell detection’ module without expansion of the nuclei to develop virtual cell boundaries. Classifiers were created to distinguish BFP^+, $NeuN^+$, and $SOX9^+$ cells, and applied sequentially. Cells containing overlapping NeuN and SOX9 were considered to be neurons (as there was a low, but detectable signal in the SOX9 channel in all nuclei with this antibody) and only cells exclusively containing SOX9 were considered astrocytes. Similar segmentation on DAPI and sequential application of classifiers were used to examine overlap between nuclear BFP, mNeonGreen, and mScarlet signal in mice shown in Fig. 2d,e.

To evaluate CREB1 levels, a representative region was selected as indicated by specific brain regions, and the nuclei were segmented on the DAPI channel as above. The measurements for the segmented nuclei were exported. The mean fluorescence intensity for the anti-Creb1 channel was obtained selected by the top 200 nuclei of highest anti-mTagBFP2 fluorescence intensity. A representative region of brain stained with secondary antibodies only was selected to determine the background mean fluorescence intensity for that channel. The same segmentation was

performed in mice injected with FLEX-GFP, with the top 2% and bottom 2% of GFP$^+$ or BFP$^+$ nuclei examined for CREB1 mean fluorescence intensity.

For mouse primary neurons transduced with AAV, live imaging was performed every other day using an ImageXpress Micro Confocal HT.ai High-Content Imaging System (Molecular Devices). The imaging chamber was warmed to 37ºC and equilibrated with 5% CO_2. The system used an Andor Zyla 4.5 camera with a Plan Apo ×10/0.45NA objective lens, an 89 North LDI laser illumination unit, 10-500 ms exposure time, 1×1 binning, and 10% laser intensity using 405-nm, 475-nm, and 555-nm lasers, running MetaXpress (version 6.7.1.157). Resulting images were imported into Cell Profiler (version 4.2.1)[46] and analyzed using a custom pipeline. hSyn1-Cre-GFP+ nuclei were segmented using the 'IdentifyPrimaryObjects' module, with expected diameter 8-40 pixels, using an Adaptive threshold (size 50) and the Minimum Cross-Entropy method, with a 1.5 smoothing scale, 1.0 correction factor, and lower- and upper-bound threshold at 0.435 and 1, respectively. Segmented objects were exported, and counted in each field, then summed across all fields within a well to calculate the number of objects per well (n=29 fields per well, n=4 wells per condition), using a custom R script. This was repeated for each timepoint. Data was normalized to fluorescent intensity at day 8 (as before that day, fluorescence intensity increased linearly with time in all channels as cells manufactured fluorescent proteins) and percentage change was calculated for each well from day 8, for subsequent timepoints through day 16.

A similar protocol was used to analyze *Rabggta* knockdown data with some modifications. hSyn1-Cre-GFP$^+$ nuclei were segmented using the 'IdentifyPrimaryObjects' module, with an expected diameter of 7-40 pixels, using an Adaptive threshold (size 50) and Minimum Cross-Entropy method, with a 1.3488 smoothing scale, 1.0 correction factor, and lower- and upper-bound threshold at 0.101 and 1, respectively. Segmented objects were exported and counted in each field, then summed across all fields within a well to calculate the number of objects per well (n = 29 fields per well, n = 3) using a custom R script. This was repeated for each timepoint. Data was normalized to fluorescent intensity at day 10 and percentage change was calculated for each well from day 10, for subsequent time points through day 26. Data was plotted using Prism GraphPad. Example images were created for Fig. 8e and 8j by importing into FIJI and applying the "red hot" LUT evenly across all images. This LUT is linear and covers the full range of the data.

Bootstrapping Analysis

To estimate the required number of mice to effectively power a screen and assess the robustness of hit detection, we performed a bootstrapping analysis using a custom Python package (`rescreener`). `crispr_screen` was used to perform differential gene abundance analyses. The analysis was conducted as follows for each mouse experiment:

1. Full Dataset Analysis: The n=12 mice of the M1 library+CaMKII-Cre and n=11 mice of the M1library+hI56i-Cre were analyzed using the `crispr_screen` tool to establish baseline results at an FDR of < 0.1, providing a list of hit genes from each full cohort

2. Bootstrapped Subset Analysis: From each cohort, multiple subsets of the treatment samples (mice) were randomly selected and analyzed:
    a. Subset sizes ranged from 1 to the total number of treatment samples (mice)
    b. For each subset size, 50 bootstrap replicates were generated by randomly sampling without replacement from the treatment samples (mice).
    c. Each bootstrapped subset was analyzed using `crispr_screen` with the same parameters as the full dataset analysis.

3. Overlap Assessment: For each bootstrap replicate, the overlap between its significant hits and those from the full cohort was calculated. Hits were defined as genes with an FDR of < 0.1.

4. Hit Recovery Analysis: For each gene identified as a hit in the full dataset analysis, we calculated the proportion of bootstrap replicates in which it was also identified as a hit. This proportion, termed the "recovery rate" was calculated as the number of times a gene was identified as a hit across all bootstraps divided by the total number of bootstrap replicates.

Supplementary Text References

39. Schnepp, B. C., Jensen, R. L., Chen, C.-L., Johnson, P. R. & Clark, K. R. Characterization of Adeno-Associated Virus Genomes Isolated from Human Tissues. *Journal of Virology* **79**, 14793–14803 (2005).
40. Penaud-Budloo, M. *et al.* Adeno-Associated Virus Vector Genomes Persist as Episomal Chromatin in Primate Muscle. *Journal of Virology* **82**, 7875–7885 (2008).
41. Wang, J.-H., Gessler, D. J., Zhan, W., Gallagher, T. L. & Gao, G. Adeno-associated virus as a delivery vector for gene therapy of human diseases. *Sig Transduct Target Ther* **9**, 1–33 (2024).
42. Yau, E. H. & Rana, T. M. Next-Generation Sequencing of Genome-Wide CRISPR Screens. *Methods Mol Biol* **1712**, 203–216 (2018).
43. Mathiowetz, A. J., Roberts, M. A., Morgens, D. W., Olzmann, J. A. & Li, Z. Protocol for performing pooled CRISPR-Cas9 loss-of-function screens. *STAR Protoc* **4**, 102201 (2023).
44. Ye, L. *et al.* In vivo CRISPR screening in CD8 T cells with AAV-Sleeping Beauty hybrid vectors identifies membrane targets for improving immunotherapy for glioblastoma. *Nat Biotechnol* **37**, 1302–1313 (2019).
45. Duan, Y. *et al.* The Clustered, Regularly Interspaced, Short Palindromic Repeats-associated Endonuclease 9 (CRISPR/Cas9)-created MDM2 T309G Mutation Enhances Vitreous-induced Expression of MDM2 and Proliferation and Survival of Cells. *J Biol Chem* **291**, 16339–16347 (2016).
46. Kabadi, A. M., Ousterout, D. G., Hilton, I. B. & Gersbach, C. A. Multiplex CRISPR/Cas9-based genome engineering from a single lentiviral vector. *Nucleic Acids Res* **42**, e147 (2014).
47. Levy, J. M. *et al.* Cytosine and adenine base editing of the brain, liver, retina, heart and skeletal muscle of mice via adeno-associated viruses. *Nat Biomed Eng* **4**, 97–110 (2020).
48. Huijbers, I. J. *et al.* Using the GEMM-ESC strategy to study gene function in mouse models. *Nat Protoc* **10**, 1755–1785 (2015).
49. Gilbert, L. A. *et al.* Genome-Scale CRISPR-Mediated Control of Gene Repression and Activation. *Cell* **159**, 647–661 (2014).
50. Bankhead, P. *et al.* QuPath: Open source software for digital pathology image analysis. *Sci Rep* **7**, 16878 (2017).
51. Stirling, D. R. *et al.* CellProfiler 4: improvements in speed, utility and usability. *BMC Bioinformatics* **22**, 433 (2021).
52. Wang, G. *et al.* Mapping a functional cancer genome atlas of tumor suppressors in mouse liver using AAV-CRISPR-mediated direct in vivo screening. *Sci Adv* **4**, eaao5508 (2018).
