# Supplementary material for: CRISPR screening by AAV episome-sequencing (CrAAVe-seq): a scalable cell-type-specific in vivo platform uncovers neuronal essential genes
Source: Nat Neurosci. 2025 Aug 22;28(10):2129–40. doi: 10.1038/s41593-025-02043-9 (PMC12497649; doi:10.1038/s41593-025-02043-9)
Supplement: Supplementary file 2 — Reporting Summary [file 41593_2025_2043_MOESM2_ESM.pdf]

nature portfolio

Corresponding author(s): Martin Kampmann
Last updated by author(s): June 13, 2025

Reporting Summary

Nature Portfolio wishes to improve the reproducibility of the work that we publish. This form provides structure for consistency and transparency in reporting. For further information on Nature Portfolio policies, see our Editorial Policies and the Editorial Policy Checklist.

Statistics

For all statistical analyses, confirm that the following items are present in the figure legend, table legend, main text, or Methods section.

n/a	Confirmed	
☐	☒	The exact sample size (*n*) for each experimental group/condition, given as a discrete number and unit of measurement
☐	☒	A statement on whether measurements were taken from distinct samples or whether the same sample was measured repeatedly
☐	☒	The statistical test(s) used AND whether they are one- or two-sided<br>*Only common tests should be described solely by name; describe more complex techniques in the Methods section.*
☒	☐	A description of all covariates tested
☐	☒	A description of any assumptions or corrections, such as tests of normality and adjustment for multiple comparisons
☐	☒	A full description of the statistical parameters including central tendency (e.g. means) or other basic estimates (e.g. regression coefficient) AND variation (e.g. standard deviation) or associated estimates of uncertainty (e.g. confidence intervals)
☐	☒	For null hypothesis testing, the test statistic (e.g. *F*, *t*, *r*) with confidence intervals, effect sizes, degrees of freedom and *P* value noted<br>*Give P values as exact values whenever suitable.*
☒	☐	For Bayesian analysis, information on the choice of priors and Markov chain Monte Carlo settings
☒	☐	For hierarchical and complex designs, identification of the appropriate level for tests and full reporting of outcomes
☒	☐	Estimates of effect sizes (e.g. Cohen's *d*, Pearson's *r*), indicating how they were calculated

Our web collection on statistics for biologists contains articles on many of the points above.

Software and code

Policy information about availability of computer code

Data collection

All software used are commercially or freely available, with corresponding literature references provided in the Methods section. Molecular Devices MetaXpress (version 6.7.1.157) or Carl Zeiss ZEN (version 2.6) were used to collect microscopy data. For qPCR data collection, we used Bio-Rad CFX Maestro (version 4.1.24.33.12.19). For dPCR collection, we used Qiagen QIAcuity Software Suite (version 2.5.0.1). For sequencing data, we used Illumina NextSeq 2000 with Illumina Basespace.

Data analysis

QuPath (version 0.4.2), GraphPad Prism 9 (version 9.5.0), Cell Profiler (version 4.2.1), Bio-Rad CFX Maestro (version 4.1.24.33.12.19), Qiagen QIAcuity Software Suite (version 2.5.0.1), ImageJ2 FIJI (version 2.9.0/1.53t). For demultiplexing sequencing data, we used llumina Dragen BCL Convert (version 3.10.4 and above).

For CRISPR screen analysis, we developed a highly efficient analysis toolkit called `sgcount` for sgRNA mapping and `crispr_screen` for differential gene abundance testing. The sgRNA mapping utility (`sgcount`, version 0.1.32) is also available on GitHub (https://github.com/noamteyssier/sgcount) and Zenodo (https://zenodo.org/doi/10.5281/zenodo.12774352)36. The differential gene abundance tool (`crispr_screen`, version 0.2.8) is also available on GitHub (https://github.com/noamteyssier/crispr_screen/) and Zenodo (https://zenodo.org/doi/10.5281/zenodo.12774208)37. All bootstrapping analyses were performed using a custom python package (`rescreener`, version 0.1.0) available on GitHub (https://github.com/noamteyssier/bootstrap_analysis_invivo_crispr_screen).

The R notebooks for analysis are available at https://kampmannlab.ucsf.edu/article/scripts-vivo-screening-manuscript. The CellProfiler pipelines are available on the Dryad data repository (DOI: 10.5061/dryad.0k6djhb9t)38 and at https://kampmannlab.ucsf.edu/article/scripts-vivo-screening-manuscript.

For manuscripts utilizing custom algorithms or software that are central to the research but not yet described in published literature, software must be made available to editors and reviewers. We strongly encourage code deposition in a community repository (e.g. GitHub). See the Nature Portfolio guidelines for submitting code & software for further information.

Data

Policy information about availability of data

All manuscripts must include a data availability statement. This statement should provide the following information, where applicable:

- Accession codes, unique identifiers, or web links for publicly available datasets
- A description of any restrictions on data availability
- For clinical datasets or third party data, please ensure that the statement adheres to our policy

All Data are available at the UCSF Dryad data repository (DOI: 10.5061/dryad.0k6djhb9t). Data from the DepMap database used to generate Extended Data Fig. 6a is publicly accessible (https://depmap.org/portal/). There are no restrictions on data availability.

Research involving human participants, their data, or biological material

Policy information about studies with human participants or human data. See also policy information about sex, gender (identity/presentation), and sexual orientation and race, ethnicity and racism.

Reporting on sex and gender	No human research participants were involved in this study.
Reporting on race, ethnicity, or other socially relevant groupings	No human research participants were involved in this study.
Population characteristics	No human research participants were involved in this study.
Recruitment	No human research participants were involved in this study.
Ethics oversight	No human research participants were involved in this study.

Note that full information on the approval of the study protocol must also be provided in the manuscript.

Field-specific reporting

Please select the one below that is the best fit for your research. If you are not sure, read the appropriate sections before making your selection.

☒ Life sciences ☐ Behavioural & social sciences ☐ Ecological, evolutionary & environmental sciences

For a reference copy of the document with all sections, see nature.com/documents/nr-reporting-summary-flat.pdf

Life sciences study design

All studies must disclose on these points even when the disclosure is negative.

Sample size	Sample sizes were determined by existing studies in the field to enable statistical analyses and reproducibility, specifically our previous CRISPRi screens in iPSC-derived neurons (Tian et al. 2019, Neuron 104:239-255). No sample size calculation was preformed.
Data exclusions	No animals or data points were excluded from the relevant analyses, with the exception of samples excluded due to poor technical quality. Poor technical quality was defined as either (1) not producing a PCR band, so were not processed for sequencing, or (2) producing fewer than 500,000 mapped reads after sequencing.
Replication	Major findings were validated using independent samples and orthogonal approaches. Numbers of replicates are listed in each figure legend.
Randomization	No randomization of samples was used since treatment group of cells were generally derived from the same population of cells.
Blinding	Blinding was not relevant to our study since no subjective rating of data was involved. Instead, we used computational quantification.

Reporting for specific materials, systems and methods

We require information from authors about some types of materials, experimental systems and methods used in many studies. Here, indicate whether each material, system or method listed is relevant to your study. If you are not sure if a list item applies to your research, read the appropriate section before selecting a response.

Materials & experimental systems

n/a	Involved in the study
☐	☒ Antibodies
☐	☒ Eukaryotic cell lines
☒	☐ Palaeontology and archaeology
☐	☒ Animals and other organisms
☒	☐ Clinical data
☒	☐ Dual use research of concern
☒	☐ Plants

Methods

n/a	Involved in the study
☒	☐ ChIP-seq
☒	☐ Flow cytometry
☒	☐ MRI-based neuroimaging

Antibodies

Antibodies used

The following primary antibodies were used:
- Rabbit anti-CREB (1:1,000 dilution, clone: 48H2, Cell Signaling Technologies, 9197)
- Rabbit anti-SOX9 (1:2,000 dilution, polyclonal, EMD Millipore, AB5535)
- Guinea pig anti-NeuN (1:500 dilution, polyclonal, Synaptic Systems, 266004)
- Alpaca FluoTag-Q anti-TagFP nanobody (1:500 dilution, clone: 1H7, Alexa647 pre-conjugated, NanoTag Biotechnologies, N0501-AF647-L)
- Rabbit anti-tRFP (1:1,000 dilution, polyclonal, Evrogen, AB233)

The following secondary antibodies were used:
- Goat anti-rabbit IgG Alexa Fluor 488 (1:1,000 dilution, Invitrogen A-11034)
- Goat anti-rabbit IgG Alexa Fluor 568 (1:1,000 dilution, Invitrogen, A-11011)
- Goat anti-rabbit IgG Alexa Fluor 647 (1:1,000 dilution, Invitrogen, A-21245)
- Goat anti-guinea pig IgG Alexa Fluor 488 (1:1,000 dilution, Invitrogen, A-11073)
- Goat anti-guinea pig IgG Alexa Fluor 647 (1:1,000 dilution, Invitrogen, A-21450)

Validation

Antibodies were validated by the manufacturers as follows:
- Rabbit anti-CREB (Cell Signaling Technologies, 9197): "The antibody does not cross-react with other ATF/CREB family members. Non-specific staining of components along the retinotectal pathway was observed by immunofluorescence in fixed frozen mouse tissue. Non-specific signal is observed in formaldehyde fixed frozen mouse retina by immunofluorescence". Source: Rabbit IgG. Applications: Western Blotting, Immunoprecipitation, Immunohistochemistry, Immunofluorescence, Flow Cytometry, Chromatin Immunoprecipitation, Cut&Run, Cut&Tag.
- Rabbit anti-SOX9 (EMD Millipore, AB5535): "Anti-Sox9 Antibody is a well characterized affinity purified Rabbit Polyclonal Antibody that reliably detects Transcription Factor Sox-9. This highly published antibody has been validated in IHC & WB." Quality systems: ISO 9001. Source: Rabbit polycolonal. Purified by affinity chromatography. Applications: Western Blotting, Immunohistochemistry, Immunofluorescence, Chromatin Immunoprecipitation, Immunocytochemistry.
- Guinea pig anti-NeuN (Synaptic Systems, 266004): Source: Guinea pig. Immunogen: "Recombinant protein corresponding to AA 1 to 97 from mouse NeuN (UniProt Id: Q8BIF2)". Applications: Immunohistochemistry, Immunofluorescence, Expansion Microscopy.
-Alpaca FluoTag-Q anti-TagFP (NanoTag Biotechnologies, N0501-AF647-L): "FluoTag®-Q anti-TagFP binds strongly to TagRFP, TagRFP657, TagBFP, mTagBFP2, mKate and mKate2." Source: Alpaca. Applications: Immunofluorescence.
- Rabbit anti-tRFP (Evrogen, AB233) : No information available.
- All secondary antibodies were highly cross-absorbed.

Eukaryotic cell lines

Policy information about cell lines and Sex and Gender in Research

Cell line source(s)

HEK293T cells were obtained from ATCC (CRL-3216) (female). MEFs were isolated from mice in our vivarium (see Methods) (mixed population of male and female mice).

Authentication

HEK293T cells were validated by supplier using STR profiling, mycoplasma testing, and bacterial and fungal testing.

Mycoplasma contamination

Cell lines were regularly tested in-house and were negative for mycoplasma using the Universal Mycoplasma Detection Kit (ATCC 30-1012KTM).

Commonly misidentified lines
(See ICLAC register)

No commonly misidentified lines were used.

Animals and other research organisms

Policy information about studies involving animals; ARRIVE guidelines recommended for reporting animal research, and Sex and Gender in Research

Laboratory animals

Mice were the only laboratory animal species used in this study. The stains used were:
- B6;129S6-Gt(ROSA)26Sortm2(CAG-cas9*/ZNF10*)Gers/J (RRID: IMSR_JAX:033066),
- B6.Cg-Igs2tm1(CAG-mCherry,-cas9/ZNF10*)Mtm/J (RRID: IMSR_JAX:030000).

	ICV injections were performed at P0-2, and mice were euthanized at 3-wk (for immunohistochemistry), 4-wk, or 6-wk of age.
Wild animals	The study did not involve wild animals.
Reporting on sex	This study analyzed male and female mice together in all experiments, except when otherwise indicated. The sex of every mouse used in this study is provided in Supplementary Table 1.
Field-collected samples	The study did not involve samples collected from the field.
Ethics oversight	All mice were maintained according to the National Institutes of Health guidelines and all procedures used in this study were approved by the UCSF Institutional Animal Care and Use Committee.

Note that full information on the approval of the study protocol must also be provided in the manuscript.

Plants

Seed stocks	N/A
Novel plant genotypes	N/A
Authentication	N/A
