# Supplementary figures and images for: CRISPR screening by AAV episome-sequencing (CrAAVe-seq): a scalable cell-type-specific in vivo platform uncovers neuronal essential genes
Source: Nat Neurosci. 2025 Aug 22;28(10):2129–40. doi: 10.1038/s41593-025-02043-9 (PMC12497649; doi:10.1038/s41593-025-02043-9)

|           | Common primer + primer a |   |   | Common primer + primer b |   |   |
|-----------|--------------------------|---|---|--------------------------|---|---|
| pAP215    | —                        | + | + | —                        | + | + |
| hSyn1-Cre | —                        | — | + | —                        | — | + |

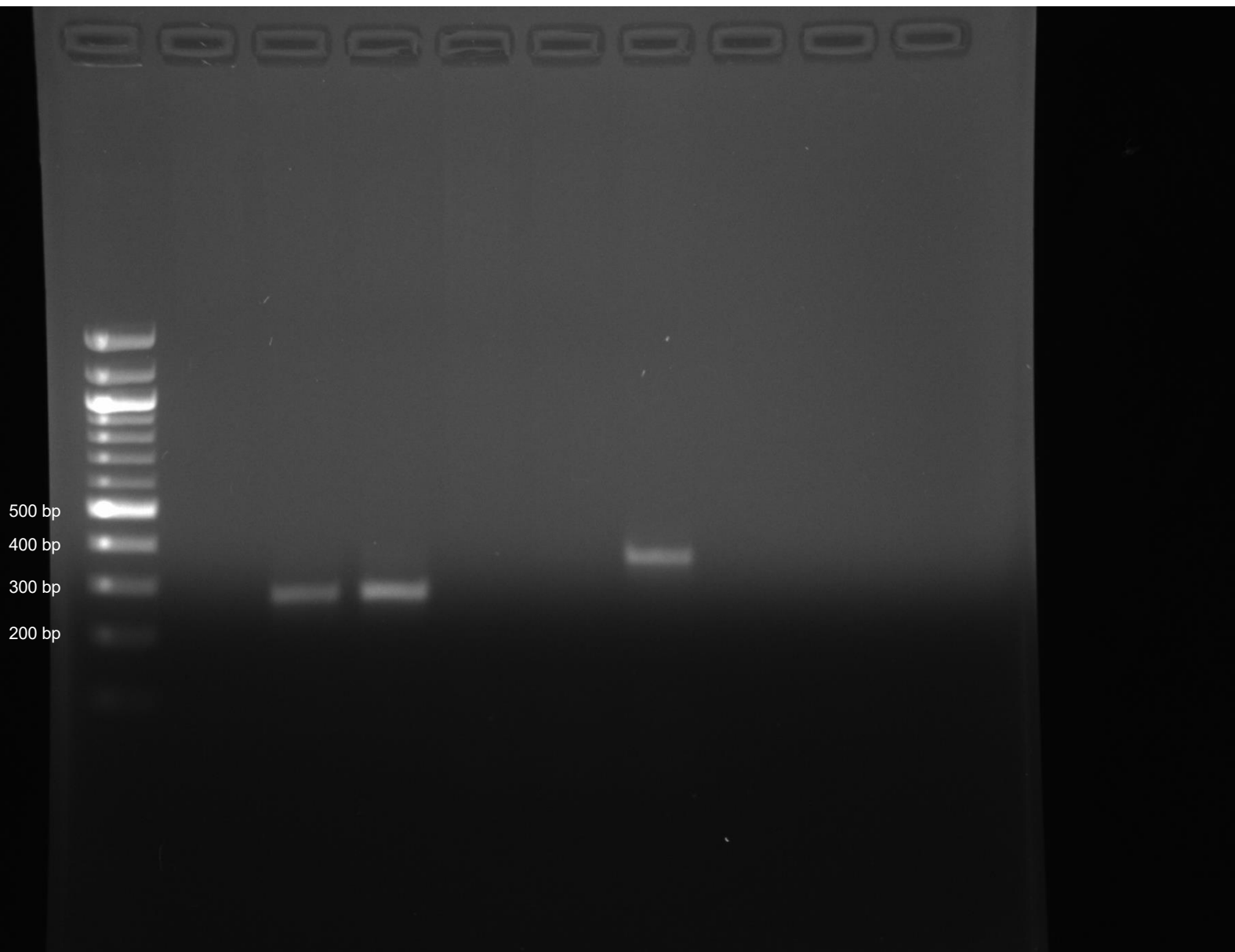

Supplement: Supplementary file 6 — Unprocessed gel from Fig. 1d. [file 41593_2025_2043_MOESM6_ESM.pdf]

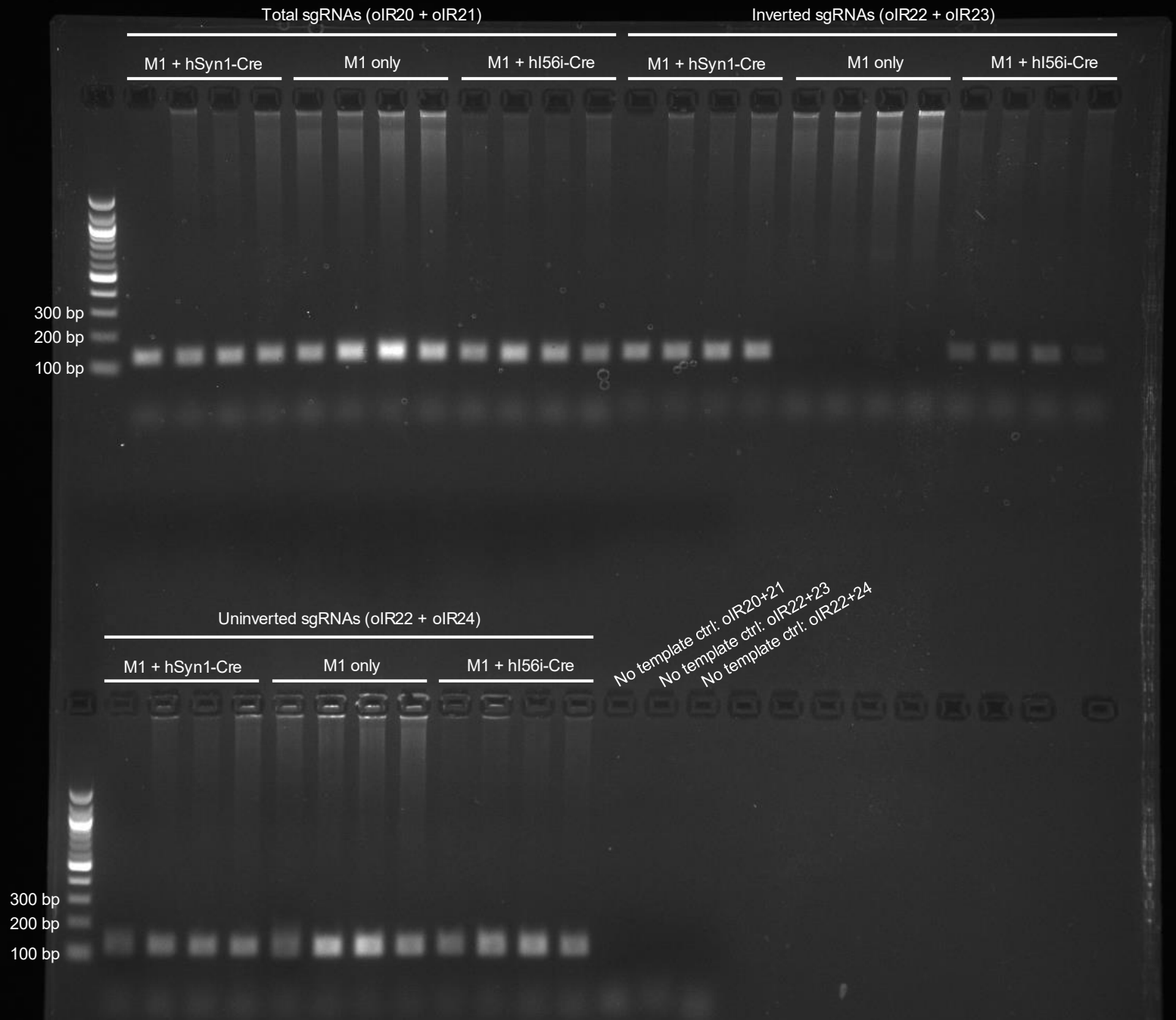

Supplement: Supplementary file 7 — Unprocessed gel from Fig. 5e. [file 41593_2025_2043_MOESM7_ESM.pdf]
